# Teleneurocritical Care (TeleNCC) Consensus Statement: The American Academy of Neurology (AAN), the American Association of Neurological Surgeons (AANS), and the Congress of Neurological Surgeons (CNS) affirm the value of this statement. Endorsed by the American Telemedicine Association (ATA).

**DOI:** 10.1007/s12028-025-02445-4

**Published:** 2026-04-06

**Authors:** Nick M. Murray, Bart M. Demaerschalk, Caitlin S. Brown, Ira Chang, Gabriel V. Fontaine, William David Freeman, Raimund Helbok, Meghan A. Knoedler, Christopher L. Kramer, Kerri L. LaRovere, Marilyn McKasson, Maranda A. Nelson, Sarah E. Nelson, Sai Praveen Haranath, Daniel B. Rubin, Syed Omar Shah, Shawn Smith, Jacqueline S. Urtecho, Chitra Venkatasubramanian, Ryan D. Williams, Craig A. Williamson, Jordan Yakoby

**Affiliations:** 1 Department of Neurosciences, Intermountain Health, 5121 Cottonwood Street, Murray, UT 84107 USA; 2 Department of Neurology and Center for Digital Health, Mayo Clinic College of Medicine and Science, Phoenix, AZ USA; 3 Mayo Clinic – Rochester, 200 First Street SW, Rochester, MN 55905 USA; 4 Carepoint Blue Sky Neurosciences, Denver, CO USA; 5 Department of Pharmacy, Intermountain Health, Salt Lake City, UT USA; 6 Departments of Neurology, Neurosurgery, and Critical Care Medicine, Mayo Clinic College of Medicine and Science, Jacksonville, FL USA; 7 Department of Neurology, Kepler University Hospital, Johannes Kepler University Linz, Linz, Austria; 8 Clinical Research Institute of Neuroscience, Johannes Kepler University Linz, Linz, Austria; 9 Department of Finance, Ascension, St. Louis, MO USA; 10 Department of Neurology and Critical Care, Mayo Clinic College of Medicine and Science, Jacksonville, FL USA; 11 Department of Neurology, Boston Children’s Hospital and Harvard Medical School, Boston, MA USA; 12 Department of Neurology, Saint George Regional Hospital, Saint George, UT USA; 13 Department of Neurology, Tufts Medical Center, Boston, MA USA; 14 Apollo Hospitals Educational and Research Foundation, Jubilee Hills, Hyderabad, India; 15 Department of Neurology, Division of Neurocritical Care, Massachusetts General Hospital, Harvard Medical School, Boston, MA USA; 16 Department of Neurosciences, Intermountain Health, Murray, USA; 17 Department of Neurological Surgery and Neurology, Division of Neurotrauma and Critical Care, Thomas Jefferson University Hospital, Philadelphia, PA USA; 18 Department of Neurology, Stanford University, Stanford, CA USA; 19 Assistant Professor in Health Care Administration, Legal Department, Mayo Clinic, Rochester, MN USA; 20 Department of Neurosurgery, Division of Neurocritical Care, University of Michigan, Ann Arbor, MI USA; 21 College of Nursing and Public Health, Adelphi University, Garden City, NY USA

**Keywords:** Teleneurocritial care, Teleneurology, Telemedicine, Neurocritical care

## Abstract

**Background and Purpose:**

Teleneurocritical care (TeleNCC) provides virtual neurocritical care in emergency departments and intensive care units that do not have continuous in-person coverage. However, there is limited evidence upon which to apply standards, benchmarks, and best practice. The scope of TeleNCC practice is beyond that of Telestroke and Telecritical Care, both of which have existing guidelines. Here, an international authorship group was convened to prepare a consensus statement on both the minimal requirements and optimal conditions for effective adult and pediatric TeleNCC.

**Methods:**

A panel consisting of 22 multidisciplinary experts in TeleNCC reviewed the available published literature, held ten virtual meetings between April 2023 and June 2024, used a modified Delphi consensus method, and voted on recommendations for best practices of TeleNCC. In two phases, a total of 13 sections on specific components of TeleNCC were written, reviewed, revised, and finalized following authorship group consensus. Specific expert rationale and applicable supportive evidence, when available, were included and integrated into each section following the recommendation(s).

**Results:**

Authorship consensus was achieved. For any disagreement exceeding 15%, there was additional group discussion and addition of explanatory text. Recommendations encompassed the following 13 sections, grouped by structure, operations, and quality; TeleNCC sections: (1) models, (2) organization in health system, (3) structural elements, (4) staffing and engagement, (5) clinical roles and responsibilities, (6) activation and communication, (7) afterhours and anticipatory guidance, (8) regulatory and credentialing, (9) finance, (10) pharmacy, (11) quality, (12) outcomes, and (13) the research gaps, training, and education. We recommend that TeleNCC: be available 24/7, though it may be utilized on a part time basis, use a reliable two-way audio-visual telecommunication system with backup options, allow for the hub TeleNCC provider to have real-time access to patient data contained in an electronic health record, as well as the original, nonprocessed raw electroencephalogram and direct diagnostic radiological imaging data. We suggest that phone-only interactions do not meet the minimum requirements for TeleNCC. The health care system should have defined roles and responsibilities for TeleNCC at the hub and spoke sites, including: a spoke site in-person provider(s) one of which is able to perform critical care procedures, clearly defined daytime and afterhours communication protocols, and have streamlined credentialing and financial arrangements. We recommend that regular continuous measurement of quality and outcome tracking is an essential component of TeleNCC practice. We review additional optimal components in well-resourced centers including the integration of advanced digital technology.

**Conclusion:**

TeleNCC may be an effective method to deliver neurocritical care in acute care environments lacking applicable in-person expertise provided the minimum requirements for standard practice and operations are met.

## Introduction

There is a paucity of Neurocritical Care Units (NCCUs) and they are often concentrated in large, urban areas. Neurocritical care by telemedicine is increasingly utilized in emergency departments (ED) and intensive care units (ICU). This is by emergent, routine, or off-hours evaluation and consultation when in-person neurocritical care expertise is not immediately available. The operations and clinical practice of teleneurocritical care (TeleNCC) is different and beyond the scope of the fields of telestroke and telecritical care (TCC), both of which have existing guidelines [1–3].

Responsibilities of TeleNCC practice are similar to that of in-person practice. These include a broad spectrum of duties, which repeat at least once daily depending on the clinical severity: (1) reviewing tests and medical records from multiple sources; (2) acquiring and/or reviewing a history; (3) performing the telemedicine clinical exam; (4) ordering neurocritical care specific medications, diagnostic tests, or procedures; 4) interpreting and communicating results to the bedside provider(s) and patient/family/caregiver independently; and (5) coordinating care with other providers/specialists (e.g., close observation locally or transfer to higher level neurologic ICU setting) [4–6].

However, for TeleNCC, there is limited evidence for best practice, standards, and safety benchmarks [4, 7, 8]. Prior studies of TCC have shown a positive effect on lower mortality and length of stay, which is reflected in TCC guidelines [9–13]. For TeleNCC specifically, development of a framework for appropriate practice is important to provide safe and effective neurocritical care to institutions and patient populations that do not have 24/7 in-person neurocritical care.

An international authorship group was convened to prepare a consensus statement on both the minimal requirements and optimal well-resourced requirements for effective and standard forms of TeleNCC, for both adult and pediatric practice. A consensus statement, rather than a guideline, was the format selected owing to the paucity of literature on this topic and the timely need for a written reference on TeleNCC to ensure standardization of high-quality and safe patient care delivery. This document contains specific recommendations and accompanying explanatory rationale for the following thirteen fundamental components of TeleNCC:

1. Models of TeleNCC
2. Organization within the healthcare system
3. Required and optional structural elements
4. Staffing and engagement of qualified multidisciplinary teams
5. Clinical roles and operational responsibilities for specific provider types
6. Activation and communication
7. Afterhours and anticipatory guidance
8. Regulatory and credentialing
9. Finance
10. Pharmacy
11. Quality
12. Outcomes
13. Research and training opportunities

The clinical domain of focus for this TeleNCC Consensus Statement is neurocritical care (NCC). Telecritical care, teleneurology, teleneurohospitalist practice, telestroke, and teleneurosurgery are all out of scope with the exception of instances of overlapping spheres of practice. The scope of this statement is also focused on developed countries with the necessary resources to provide two-way audio–video telemedicine interactions for clinical care. For any developing countries with limitations to telemedicine or barriers to access to advanced neurodiagnostic and therapeutic modalities typical to neurocritical care (NCC) [14], these recommendations may be beyond scope.

Finally, the terminology used to refer to location of patients and where the TeleNCC provider(s) are located are not uniformly used within the literature, hence definitions specific for this document are as follows. Both telestroke and TeleNCC networks consist of originating sites where the patients are located and distant sites where the TeleNCC provider is located [2]. Most TeleNCC systems exist as either a distributed or a hub-and-spoke model, or less commonly, as a single center model with the same provider delivering night virtual coverage or an eICU model of central site covering many other locations on a supplemental as needed model.

In the distributed model, TeleNCC services are delivered to hospitals from providers at distant sites on a contractual basis. The neurointensivist (a physician provider with residency and fellowship training and board-certification or board-eligibility in neurocritical care) may have no other connection with the hospital besides the remote critical care discrete episode. If a patient requires a higher level of care, protocols are usually established to facilitate transfer to a nearby neurocritical care center. Coverage for neurocritical care consults may be supplied by an organized group of providers or an independent for-profit company.

In the hub-and-spoke model, a neurocritical care center, such as an academic medical center, provides TeleNCC services at the site distant to hospitals within its catchment area (originating sites). When transfers are necessary, the hub center receives the patient from the spoke hospital, having already observed and evaluated the patient by telemedicine. In most cases, the hub hospital is the closest neurocritical care center to the spoke hospital. For simplicity, we will consistently refer to “hub” site in any instance we are referring to a hub site and a distant site where the TeleNCC provider is located; and finally, to a “spoke” site in any instance where we are referring to a spoke site as the originating site where the patient is located. We recognize that there is insufficient evidence to fully substantiate best practices for either Hub-Spoke or Distributed models of acute care telemedicine, therefore both of these most common forms are presented.

## Methods

This work was approved by the Neurocritical Care Society (NCS) Board of Directors and Guidelines Committee. The NCS Guidelines Committee tasked two chairs (N.M., B.D.) to form a writing panel of 22 international experts in TeleNCC to develop the consensus statement. Beginning April 2023, the panel drafted 13 main sections each with subsections focused on questions relevant to practice of TeleNCC. The chairs assigned two writing panelists to each of the sections, as primary and secondary authors. The panelists performed comprehensive independent literature searches to inform their recommendations. All available literature up until 1 January 2024 was included for consideration in the panel’s consensus and voting. Both the writing group authors as well as two independent medical librarians performed searches. Search terms pertaining to telemedicine, telehealth, teleneurology, telestroke, eICU, remote, virtual, neurocritical care, telecritical care, neurotelecritical care, and teleneurocritical care with all permutations using AND / OR as well as hyphenation in EMBASE, MEDLINE, SCOPUS, Web of Science, PubMed, and Google Scholar were used.

Recommendations were collectively derived from: published primary literature, consensus of expert opinion (detailed below), as well as from the recommendations for operational standards, documentation, and computerized interfaces for telecritical care outlined in the AHA/ASA and American Telemedicine Association (ATA) guidelines for telestroke and telecritical care [1, 2].

**Fig. 1:**
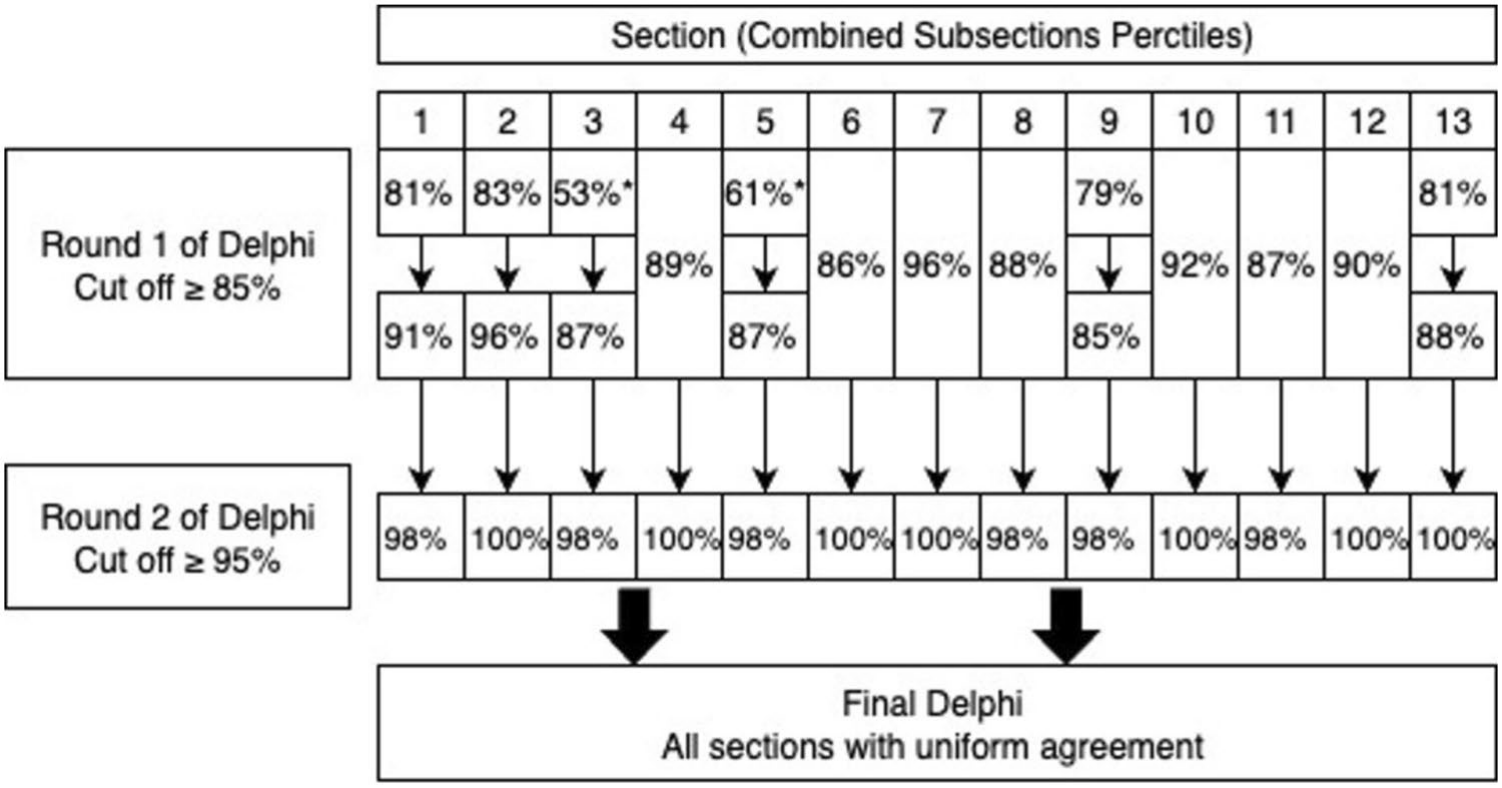
Modified Delphi consensus rounds. Each Topical Section is comprised of the average subsection percentile’s agreement. In round one, sections with disagreement > 25% or exceptionally compelling reasons from one or more members had multiple distinct discussions and voting, as designated by an asterisk (*). Some examples include: strong consensus for necessity of two-way audio-visual for consultations, training requirements, responsibilities for advanced practice clinicians, and avoidance of specificities, such as staffing ratios. The final consensus percentile agreement is represented as the second percentage in column. In round two, modifications were made until uniformity in agreement, to then allow complete agreement at the end of each section’s voting round

The writing panel met in person and via teleconference on 17 August 2023, in Phoenix Arizona; however, the remaining meetings were entirely virtual. Voting using a modified Delphi consensus took place via two rounds each for the first phase of sections and the second phase of sections. The first round of each voting set was cast via an electronic online form. Any disagreement by 15% or more of panelists or any single strong disagreement was then discussed in subsequent virtual meetings. Modification and repeated voting occurred until the consensus of 95% panelists was obtained (Fig. 1). A final discussion and consensus voting took place for the completed TeleNCC statement document.

Internal experts within the Neurocritical Care Society and external stakeholders then reviewed the final guideline draft.

For a brief reference of all condensed sections one through 13 and their recommendations, please see the last table, Table 8.

## Section 1: TeleNCC Models

### Question 1.1: Which TeleNCC Models Meet Standard of Care Delivery for Replacing In-Person Neurocritical Care?

#### Recommendation 1.1:

The panelists suggest that the minimum TeleNCC model should be neurointensivist-directed and incorporate in-patient care interactions similar to that of an in-person neurointensivist (minus the physical interactions and interventions). This often takes the form of a hub-and-spoke model. The hub provider for spoke TeleNCC sites provides consultative decision-support via (a) telemedicine components and (b) access to original unaltered or processed raw medical data necessary for high level patient care, similar to an in-person provider (*Details in TeleNCC Structure, *Sect. 3*).* We recommend that TeleNCC coverage can be offered by default as 24/7/365 continuous; however, it is reasonable for some health systems to make use of only off hours coverage (i.e., nights and/or weekends), so long as there are clear time-based coverage constructs of roles and responsibilities (*Expected availability of TeleNCC services are further detailed in TeleNCC Structure, *Sect. 3). This would include single center models in which a NCC provider provides qualified TeleNCC care while physically out of the hospital.

The hub provider, or collaborating provider if an APP or fellow is providing TeleNCC, should be board certified or board eligible in NCC (*Further details in the Roles and Responsibilities, *Sect. 5), be available for routine rounding and responding to emergent consultations, document in the spoke EHR, and adhere to the latest evidence-based and guideline-recommended disease diagnostics and management. *Further details on these items in the TeleNCC Structure *Sect. 3*.*

Some forms of practice do not meet minimum criteria for TeleNCC. These include telephone only consultation, monitoring of neurological data feeds only without a standard practice for interactive care, or consultation without access to raw neurological data that would be typically required for critical care decision-making. In some cases, there may be limited access to external data at the spoke site; however, if there is adequate two-way audio–visual capacity for a standard consultation, this may be permissible to ensure provision of care to less resourced settings.

Finally, the panelists came to uniform consensus that TeleNCC would not be able to safely provide care as the primary (i.e., in-person) provider for an ICU patient, given the need for the primary provider to have physical presence for procedures, proximity, and time for hyper-emergent response (*Further details in the Roles and Responsibilities, *Sect. 5). Importantly, the same provider performing TeleNCC could be the primary provider in other contexts, as NCC training, board certification, and the current practice reflects providers in primary and or consultant roles. In addition, in some TeleNCC models, this provider can be and is providing comprehensive critical care for all organ systems, not only neurological, for the spoke patient.

#### Rationale 1.1:

The writing panelists supported an Intensivist Directed Model for providing TeleNCC that is based on in-person neurocritical care unit models [8, 14]. This model allows for organizational flexibility while still maintaining clearly defined structural elements.

The panelists recommended for both hub and spoke sites that there should be mutual discussion and agreement over the scope and intensity of TeleNCC interactions. This encompasses time to response, frequency of rounding, pathologies of coverage, and scope of practice (*Additional details in Role and Responsibilities, *Sect. 5)*.* The hub TeleNCC provider should be able to provide the highest level of NCC care, as able based on the spoke’s staffing, resources, and equipment parameters. The level of care provided at the spoke should be listed as similar to that of in-person NCCUs with modifications as necessary for the NCC provider being off site via virtual telemedicine, as described previously [14].

Different coverage models for NCC have been described in the literature. Importantly, Moheet et al. developed neurocritical care unit standards including ideal neurocritical care unit organization, available hospital services, and training. Specifically, these authors specified different levels of neurocritical care units: level I care for complex neurological patients who may require advanced therapies and possess advanced monitoring capabilities and training programs; level II can safely manage patients with stable neurocritical care conditions; level III can stabilize patients and then facilitate transfer to level I or II units (Direct link: https://pubmed.ncbi.nlm.nih.gov/30251072/). Modifying these as appropriate so that they can be used broadly in a variety of TeleNCC models seems prudent; given TeleNCC models’ current nonuniformity, each unit would ideally conform to one of these levels depending on a given hospital’s capabilities.

## Section 2: TeleNCC Organization Structure Within the Healthcare System

### Question 2.1: What are the Core Operational Components for a TeleNCC Needs Assessment and What Service Gaps are Commonly Identified in a Typical Hub and Spoke Healthcare System?

#### Recommendation 2.1:

A needs assessment for TeleNCC in a healthcare system should encompass the characteristics of coverage. Coverage models may vary according to need and include temporary coverage gaps or short term, permanent, or variable hours to accommodate a spectrum of patient volumes. The degree of operational planning and startup will be distinctly different depending on whether there is existing NCC, acute teleneurology consultation, or telestroke infrastructure or if the coverage is de novo. Knowing the type of coverage can establish TeleNCC roll out expectations and the timeline. Furthermore, a needs assessment and/or feasibility questionnaire should be performed within the neurology service line and other stakeholder service lines outside of neurology that would work with TeleNCC to advise the organizational structure. This includes emergency medicine, hospitalists, neurosurgery, interventional neuroradiology (i.e., endovascular interventionalist), critical care, and neuroradiology.

The coverage should incorporate the parameters specified in the above needs assessment as well as those desired for meeting or exceeding the defined level of NCC coverage, Level 1–3, as described previously [14]. The healthcare system’s vision for the future of critical care neurological disease should be assessed and defined to best plan for growth and evolution. In parallel, the health system receiving TeleNCC may wish to identify a distinct, dedicated TeleNCC administrative unit and financial support, with oversight by dedicated physician, nursing and hospital administrative executive leadership.

#### Rationale 2.1:

Setting up a TeleNCC service at any given hospital requires an understanding of the goals of the hospital system in delivering this level of care. There are many questions that should be asked to understand the underlying driving forces guiding the organization of the service line.

Goals may extend from increasing neurological patient complexity, i.e. achieving comprehensive stroke center or thrombectomy capable stroke center status, or increasing patient volume, increasing outreach opportunities, instituting gap coverage, providing supplemental coverage, providing optimal postprocedural care for neurosurgery and neuro-interventional radiology [15, 16]. Building infrastructure for an in-person neurocritical care center may take considerable time and resources and may not be achievable for every local health care system. TeleNCC may fill gaps during planning phases and prior to implementation [14].

Sample questions for assessing healthcare system needs may include:

1. Is there an existing in-person neurocritical care unit and what level of service does it provide?
2. If there is an in-person or telemedicine model already in existence, what are the gaps observed in the current staffing and daily coverage model?
3. Is TeleNCC a temporary solution for gap coverage or a new enduring line of service?
4. What hours of coverage is the TeleNCC service providing?
5. With what department(s) will the TeleNCC service be aligned and complement—neurosurgery, neurology, critical care (medical-surgical), trauma, cardiac critical care, transplant critical care, etc.?
6. What extent of medical critical care services in addition to neurocritical care services are to be provided by TeleNCC?
7. What professional classifications and what qualifications will the onsite in-person partner to TeleNCC have?

The health system’s planning for the level of NCC care is similarly important, specifically, to which tier of NCC level is desired. As described previously for in person NCC, but not for TeleNCC, level I–III units exist [14], with level I units hosting the highest level of NCC:

- Level I units provide the most comprehensive neurocritical care for patients with the most complex neurological emergencies who require advanced interventions. These units provide definitive and expert care to a wide variety of neurocritical care disorders. These units offer a full complement of advanced monitoring, surgical and medical treatments, have the capability to provide physician fellowship and advanced practice professional training.
- Level II units stabilize and safely manage stable neurocritical disease processes, while having established relationships with level I neurocritical care units.
- Level III units provide emergent evaluation and stabilization of patients presenting with neurological emergencies and facilitate transfer to level I and II units when appropriate.

Certification of TeleNCC Level units may be developed and offered.

### Question 2.2: What are the Partnerships and Service Line Alignments that are Needed for a TeleNCC Service?

#### Recommendation 2.2:

Well-defined department roles and staffing partnerships are necessary for TeleNCC service function, in particular the on-site ICU staff and the non-ICU neurology staff. This similarly applies to the ED. Specific scope of coverage for each provider should be defined, especially for covering non-neurological medical issues, procedures, and care before and after the ICU admission. Comanagement team roles should be identified and agreed upon, as the needs will be different for individual health systems. This is particularly important if the ICU is closed or open in a primary patient provider structure. Explicit coordination with vascular neurology, neurosurgery, and endovascular specialists should be done. 

In parallel with these service line partnerships, the financial coverage and distinct routes of cost benefit provision need to be mapped for the health system. Further details are addressed in the *Finance Section, Number 9*.

#### Rationale 2.2:

Introduction and integration of a TeleNCC service within an existing ED or ICU model can be complex and differs on the basis of the structure and needs of the institution. To ensure that valued, nonduplicative work, and comprehensive care is provided it is important that core roles and expectations are defined, including:

- Non-neurological medical management
- Division of labor between TeleNCC and non-ICU neurologist teams, including vascular neurology, neurosurgery, and endovascular teams, whether in-person or virtual
- Admitting, consulting, discharging, and patient follow-up responsibilities
- Handoff process between providers upon patient location changes
- Operations oversight and monitoring, local leadership structure
- Bedside collaborative partner identified for specific tasks: examination, order entry, procedures, family conversations or goals of care meetings
- Billing relationship based on involvement of local team

Standardized roles, responsibilities, and checklists can facilitate clinical performance and the highest efficacy of collaborative care [14, 17, 18]. Adherence to such ensures adherence to published performance metrics from the ASA, American Academy of Neurology, and The Joint Commission.

### Question 2.3: What are the Best Measures of TeleNCC Operational Quality, Success, and Volume?

#### Recommendation 2.3:

The TeleNCC service or the host hub site should capture and track metrics for both system impact as well as for patient care and outcomes. Assessment of consult volume, rapid access to NCC care, inpatient stay characteristics, discharge disposition, and extended follow-up functional outcomes, as well as cost breakdowns with TeleNCC care is important for assessment of effect and sustainability of the TeleNCC service. The effect on the system, inclusive and beyond volume and quality, should be explored, from changing procedural volumes, transfer rates, provider satisfaction, and adherence to established benchmarks for the given level of NCC. The system should strive to capture adherence to defined performance and treatment metrics for specific core neurocritical disease states, including large vessel occlusive stroke, spontaneous intracranial hemorrhage (ICH), aneurysmal subarachnoid hemorrhage (aSAH), and traumatic brain injury (TBI). For patients, the operational success of TeleNCC can be measured by patient satisfaction of the virtual experience, quality of life outcomes at discharge and beyond, and readmission rate.

#### Rationale 2.3:

Monitoring and tracking the impact and efficacy of TeleNCC is necessary for a high-performing operational system. This may take the form of real time automated metric capture and assessment or semi-regular data extraction and review. Doing such tasks ensures that the value of the system for all stakeholders is favorable and that underperforming aspects may be addressed.

The impact on the inpatient stay and longitudinal outcomes for individual disease states are important, in particular when compared over time and between TeleNCC and non-NCC involvement. The differences, if any, of these two forms of neurocritical care can be used to develop improved operational approaches for patient care.

*Quality metrics and assurance specific to TeleNCC are further discussed in the Quality *Sect. 11.

## Section 3. TeleNCC Organization—Minimum Required and the Optional Structural Elements

### Question 3.1:

#### Sub-Section 3.1A: What are the Minimum Required Structural Elements for the Two-Way Audio Visual TeleNCC Interactive Consultation?

#### Recommendation:

As described in the *TeleNCC Models *Sect. 1, an audio-only telephone consultation and/or monitoring of neurological data (vital signs, electroencephalogram (EEG), etc.), do not meet the minimum criteria for TeleNCC. TeleNCC must meet or exceed the benchmarks (below) for: telemedicine technology interface, involved personal, core components of the consultation, and documentation.

1. Telecommunication infrastructure must have reliable high-speed internet connectivity for videoconferencing, data transmission, and remote monitoring [5]. Both hub and spoke institutions should have these bandwidth and network capabilities. The videoconferencing platform should be secure and HIPAA-compliant for real-time two-way interaction. It should have reliable webcams, microphones, and speakers or headsets. Monitoring systems that relay neurological data to the TeleNCC hub or those into which a hub provider can remotely connect may be continuous or intermittent depending on the data type. These include vital signs, output from intracranial monitors, EEG, and radiologic studies. The system should be capable of transmitting all physiological and diagnostic data for analysis in real-time, on demand, or by alert generated from the local bedside nurse to the TeleNCC team. The TeleNCC team should be informed of or able to view the data as needed during the care without having to activate or request separate data transfer pushes or processes. At a minimum, easy access to the raw neuroimaging and EEG data is necessary. Finally, in the case of technical failure, downtime procedures should be developed.
2. Advanced imaging capabilities should allow radiologic studies to be viewable by the hub provider via a remote portal or be integrated into the hub PACS. TeleNCC providers should easily be able to access and assess relevant neuroimaging studies during the consultation.
3. Electronic health record (EHR) integration should allow seamless access to medical history, imaging studies, laboratory results, and other relevant clinical information. This may be via the same EHR at the hub and spoke site, or allowance of the hub provider to remotely log into the spoke EHR.
4. Data security and privacy measures must be in place to ensure integrity and protection of patient health information (PHI). These should adhere to relevant local and national regulations, such as the Health Insurance Portability and Accountability Act (HIPAA) in the USA.
5. Trained personnel for providing hub TeleNCC encompasses, at a minimum, neurocritical care physicians; additional providers are often involved in the optimal setting: advanced practice providers, nurses, pharmacists, and technical support staff who are proficient in using the telemedicine technology [5, 14]. The spoke team must have an onsite clinical provider who can effectively collaborate remotely with the hub team. *Staffing, training standards, and credentials are further defined in the Staffing *Sect. 4. *Methods of activation as well as collaborative roles and responsibilities of providers involved in the care are described in the Daily Clinical Operations and Activation and Communication Sects. 5 and 6, respectively.* General guiding telemedicine privileges for hub TeleNCC providers are presented in Table 1.
6. Clinical documentation should be clear, concise and accurate as required for patient care, communication, and legal compliance. Documentation must adhere to relevant legal and regulatory requirements for telemedicine, including informed consent, privacy and security measures. Institutions should follow their local policies and guidelines for clinical documentation in telemedicine. The key elements that should be included in the documentation of TeleNCC consultations are summarized in Table 2 and a sample TeleNCC clinical note template is shown in the **Supplementary Document, as Fig. 1**. Many patients will not be able to complete the full neurological exam as listed, hence focus on the comatose-specific elements may be required.

**Table 1:** Common teleprivilege summary for teleneurocritical care (TeleNCC) providers in the evaluation, treatment and guidance of procedures involving neurocritical care patients

*Teleprivileges*
Remote access to patient records in the EHR
Real-time video telemedicine consultations with providers, patients, parents/families/caregivers
Review vital signs, diagnostic imaging, and laboratory results
Evaluations and treatments based on information provided through remote monitoring systems and the electronic health record
*Privileges for provider evaluations and treatments*
New neurocritical care consult in the ED or ICU at partnering institutions
Evaluation or treatment of any acute and life-threatening neurological and neurosurgical condition (e.g., traumatic brain injury, ischemic stroke, intracranial hemorrhage, seizure/status epilepticus, acute CNS infection/demyelination, neoplasm, hydrocephalus, acute myelopathy, neuromuscular weakness, neurologic complications of systemic illness, hypoxic ischemic brain injury, encephalopathy/delirium, and coma) in the ICU
Evaluate and treat common perioperative neurosurgical and neurointerventional conditions in the ICU
History, vital signs, and/or physical examination with concerning findings
Document clinical decision- making and decisions about end-of-life care in the electronic health record
Decision* to administer thrombolysis or recommend consideration of endovascular thrombectomy (mechanical thrombectomy), decompressive craniectomy, or other advanced neurological interventions
Decision* to provide therapeutic hypothermia
EEG or amplitude EEG monitoring and interpretation*
Interpretation* of transcranial doppler ultrasonography
Routine medication management
Test ordering (e.g., EEG, head CT, brain MRI) and interpretation of results for subsequent clinical management
Participation in family and team meetings for a neurocritical care patient
Decision* to transfer a neurocritical care patient between levels of care or between institutions
*Guidance for procedures*
Supervision* of brain death examination^#^
Supervision* and guidance of lumbar puncture^#^

*CNS* central nervous system, *CT* computed tomography, *EEG* electroencephalogram, *ICU* intensive care unit*, MRI* magnetic resonance imaging

*Signify that these items are generally done in a collaborative manner with the local treatment team or the attending of record for the study itself. These are additionally several specific practice types that are less commonly part of core privileging sets

#Advise that the provider documents their two-way audio-–visual interaction pertaining to their assistive role, and its limitations implied by the telehealth interaction

**Table 2:** Key elements for documentation of TeleNCC consultation

Patient information—name, date of birth, medical record number
Date, time, and location of the telemedicine consultation at patient’s geographic location
Consulting TeleNCC provider—name, credentials, contact information, and location
Referring provider—provider name, credentials, contact information, and geographic location
Reason for consultation—chief complaint, medical history, and relevant diagnostic findings
Medical history—concise summary of the patient’s medical history, relevant comorbidities, previous neurological conditions and medications
Neurological assessment—comprehensive neurological assessment, including any focal deficits utilizing applicable scoring systems (e.g. National Institutes of Health Stroke Scale, ICH score, Hunt Hess Score, Mayo Clinic FOUR Score, Glasgow Coma Scale, etc.)
Diagnostic data—relevant tests such as CT scans, MRI, EEG, or lab results. Interpret these findings in the context of the patient’s condition
Treatment plan—outline the proposed treatment plan, medications, procedures or interventions recommended for each neurological problem
Rationale—explain the rationale behind the treatment plan and how it addresses the patient’s specific neurological condition
Discussion and communication—note any discussions held during the telemedicine consultation, including communication with the patient, their family, or other healthcare providers. Document any recommendations or decisions made. It is reasonable to include prognostic implications of the discussion that may facilitate the spoke providers’ understanding, inpatient expectations, and disposition planning
Follow-up and contingency Plans—specify any necessary follow-up actions, such as additional consultations, further diagnostic tests, or changes to the treatment plan. This may include “if, then” statements for implementation of contingency plans for expected results, exam changes, etc
Informed Consent—document that the patient or their legal representatives provided informed consent for the TeleNCC consult, including the risks and benefits of telemedicine and any charges that may apply (if applicable and if not already covered in healthcare system registration)
Provider’s Signature and Attestation—sign and date the consult note and provide attestation
Coding and Billing information—if applicable, include the appropriate medical codes for billing purposes

### Question Sub-Section 3.1B: What Additional TeleNCC-Specific Structural Elements May be Included in the Optimal Setting(s)?

#### Recommendation 3.1B:

In well-resourced settings additional, advanced TeleNCC components could be considered. These include dedicated staffing: a spoke TeleNCC APP and/or RN who have assigned hours or percentage of FTE to TeleNCC. In parallel with staffing, it is optimal to have a backup two-way audiovisual cart or robot or other alternative communication device in case of primary technology failure. Some devices have functionality for remote physical movement and interaction, which can be beneficial in case of limited staffing at the spoke site [19–21]. Incorporating evidence-based and regulatory body approved decision support modalities and technology may be considered. This may include some but not all types of artificial intelligence and automated algorithms within and outside of the telemedicine platform.

Use of mobile health data applications offer additional flexibility and accessibility.

Collaborative platforms, such as shared workspaces or virtual whiteboards, enable multidisciplinary teamwork and information sharing. Establishing education and training programs for all healthcare professionals involved in TeleNCC ensures proficiency in using the technology and staying up-to-date with best practices. *Further details in Research and Training *Sect. 13*.*

#### Rationale 3.1:

The recommendations for minimum requirements are considered to be central to the development of any TeleNCC model because they are feasible, sufficiently broad, and basic in scope. The infrastructure for TeleNCC systems should therefore enable telecommunications that focus on providing timely, safe, and effective critical care services to patients in the ICU with life-threatening conditions involving the nervous system and allow collaboration between neurocritical care teams at local and partnering institutions regardless of geographical barriers. Readily available, real-time access to patient records, radiologic and neurological data is integral to management of neurocritical care patients. There was a uniform group consensus that access to the EHR and the raw images are necessary to provide the minimum standard of neurocritical care via telemedicine.

The optional components for well-resourced settings may enhance the effectiveness and scope of TeleNCC beyond the minimum requirements. Examples of core components of the TeleNCC interaction and documentation, for some but not all forms of TeleNCC, are summarized in Tables 1 and 2.

Of note, TeleNCC models differ across countries, healthcare systems, systems of care, and patient needs, and no single model has been extensively evaluated for effectiveness *(Details in TeleNCC Models *Sect. 1). In addition, while some aspects of the structure of TeleNCC models are likely to be similar to telestroke models, the remote care of ICU patients is more complex. TeleNCC patients have a variety of clinical neurological emergencies, and need close continuous 24/7 acute care, which requires additional critical care effort and time [4–6, 22].

## Section 4: Staffing and Engagement of Qualified Multidisciplinary Hub and Virtual TeleNCC Teams

### Question 4.1: What are Appropriate Levels of Staffing and Engagement for Providers of Teleneurocritical Care Services (Hub Providers)?

#### Recommendations 4.1:

Staffing should be provided by qualified TeleNCC care providers, as demonstrated by applicable residency and fellowship training and board-certification or board-eligibility in neurocritical care for physicians, or appropriate training and credentialing for other types of providers (*Specified in *Sect. 5*, Roles and Responsibilities*). A qualified medical director may be assigned to supervise TeleNCC care staffing and clinical care. The medical director oversees provider training and is responsible for provider engagement, quality improvement, and research activities.

It may also be helpful to appoint a staff member with TeleNCC program manager duties, to provide overall administrative oversight of the program. Working in conjunction with the medical director, the program manager may serve as the primary point-of-contact with credentialing, IT, scheduling, and legal offices at both hub and spoke sites, ensure that current credentialing and contracts are in place at spoke sites, oversee scheduling, billing and coding practices, tracking of metrics, and quality assurance procedures.

An up-to-date coverage schedule for TeleNCC care providers should be maintained and shared with all participating sites. Ideally, a system should be developed to ensure that a backup provider is available who can take over for the assigned provider in the event of illness or equipment failure. The medical director may also develop procedures by which a back-up provider is activated when clinical volume exceeds a single provider’s capacity.

TeleNCC providers should receive adequate training in the use of telemedicine and telecommunication devices. Dedicated IT support professionals who can troubleshoot technical difficulties should be available at all times. TeleNCC providers should consider participating in TeleNCC research, quality improvement activities and regular quality assurance meetings, ideally in conjunction with local site team members.

Finally, the authorship group did not reach consensus on specific staffing ratios of providers to patients. This was owing to program variability in scope, location, disease severity, as well as the international differences as well as potential legal implications of such specificity.

#### Rationale 4.1:

A variety of staffing models for TeleNCC providers exist. The most common is the traditional hub and spoke model that is widely used in telestroke, where providers affiliated with a single neurocritical care hub, frequently a tertiary or quaternary referral center containing a level I neurocritical care unit, provide TeleNCC care services to several outlying medical centers, i.e., spokes [14]. In this model, TeleNCC care providers frequently provide continuous coverage via routine daily examinations and evaluation (E.g., NCC rounds) on patients in addition to maintaining availability for consultation on an as-needed basis.

Decentralized program structures also exist where a remote neurointensivist is available to see patients at a local site, often activated by alarms or local providers on an as-needed basis, or to provide subspecialty consultative services to support overnight trainees and mid-level providers [1, 3, 8, 13]. TeleNCC providers may be housed in a single centralized telehealth facility, though they most often utilize computers equipped with audiovisual telemedicine equipment to monitor and evaluate patients from private location(s) of convenience, such as home or office. At present, peer-reviewed research has not demonstrated the superiority of any single staffing or coverage models.

Regardless of the TeleNCC staffing model being used, it is essential that TeleNCC care providers have clearly defined roles, responsibilities and leadership structure. To that end, a TeleNCC care medical director should be appointed to oversee the program and its operations, though that person may choose to delegate some administrative tasks to others. The medical director will oversee TeleNCC care provider training and establish procedures for quality assurance through regular interactions with both TeleNCC care providers and remote site staff members. The directors should establish and maintain provider scheduling, develop contingency plans for unexpected coverage gaps, and engage in hospital service line and programmatic meetings. The director should consider participation in quality improvement activities and provide feedback on quality metrics and quality assurance benchmarks. TeleNCC providers should be encouraged to participate in continuous medical education (CME) activities and/or research applicable to TeleNCC. The TeleNCC director may hire staff to assist with data collection and research. At larger programs, it may be necessary to appoint a program manager who can assist the medical director by providing overall administrative oversight.

Continuously functioning telecommunication infrastructure is essential to provide neurocritical care services. Consequently, it is essential that IT staffing resources are available to support TeleNCC providers and quickly repair or replace faulty technology that could interrupt the delivery of clinical services.

### Question 4.2: What are Appropriate Levels of Staffing and Engagement for Participating Sites Receiving TeleNCC Services (Spoke Sites)?

#### Recommendation 4.2:

Each participating site should have a single dedicated individual (site champion) who is responsible for providing training and supervision for other medical providers and staff members utilizing TeleNCC services. The site champion should also work jointly with the TeleNCC medical director to monitor quality of care and relevant metrics at their site.

In an optimal setting, the spoke site would have a nonprimary team, neurocritical care-focused nurse practitioner (NP) or physician assistant (PA) provider(s) dedicated with some portion of their time to TeleNCC rounds and clinical care. These providers should have completed the necessary training and credentialing, as described in Sect. 5*, Roles and Responsibilities*.

At a minimum, providers and nurses who interface with TeleNCC providers at participating sites should receive adequate training in the relevant telemedicine and telecommunication technologies, TeleNCC workflows, policies and procedures, and key clinical assessments. A provider capable of performing bedside critical care procedures, such as airway management and central line placement, and responding to emergencies in-person must be available at each participating site.

In the optimal setting, neurocritical care specific collaborative teams should be locally available and readily interfaced with the TeleNCC team. These include vascular neurology, electrophysiology and neuromuscular neurology, hospital neurology (neurohospitalist), epilepsy and EEG technologists, neurosurgery, and neurointerventional radiology. Providers and staff at participating sites should be encouraged to participate in medical education conferences, quality assurance and research activities in conjunction with TeleNCC providers at hub sites.

#### Rationale 4.2:

There are several different models by which medical team members at spoke sites engage with TeleNCC providers. At many sites, a single provider or small group of providers, often an APP (s), participate in daily rounds, examine patients, and develop the treatment plan in collaboration with the TeleNCC provider. Sometimes spoke intensivists or hospital neurologists will jointly round and/or examine patients with the teleneurointensivist, or the bedside registered nurse (RN) will be the primary collaborator and point of contact with the TeleNCC provider. Respiratory therapists (RTs), case managers, speech, physical and occupational therapists may also directly interface with the TeleNCC service.

To date, there are no published studies examining the effect of spoke site staffing models and training on TeleNCC patient outcomes. As with TeleNCC provider staffing models, the optimal arrangement will likely vary from site to site on the basis of available personnel and resources. The well-described volume–outcome correlation supports the development of a core spoke team who has a focus or additional training on neurological patient care. By having a small team of providers, such as neurology or critical care APPs dedicated to collaborating with TeleNCC providers and implementing recommendations, there is better communication and patient care [23].

However, many spoke sites lack resources to dedicate a focused, core team to the TeleNCC service. In this situation, many different members of the medical team (RNs, residents, fellows, APPs, neurologists, intensivists) may be the primary care staff contact depending on the circumstances. Therefore, because spoke site providers play a vital role activating telemedicine technology and communicating with TeleNCC providers, it is important that they receive adequate training in technology, protocols, and activation procedures relevant to TeleNCC, as well as best practices in recognizing and evaluating neurological emergencies. A training program such as Emergency Neurological Life Support (ENLS), may be particularly helpful in this regard.

Regardless of the spoke site staffing model, it is essential that at least one TeleNCC site champion be assigned to supervise the development of TeleNCC workflows, policies, procedures and ensure the provision of adequate training to the local site staff who works with teleneurointensivists. The site manager should regularly interface with the overall TeleNCC program director and, if applicable, program manager to ensure the delivery of high-quality care. The site leader should be encouraged to participate in joint research activities and conferences, while encouraging other members of the site medical team to participate in such activities. While TeleNCC providers make important contributions to patient care and in many models are available to quickly evaluate a patient in an emergency, they rely on spoke site providers possessing adequate training and availability to perform essential critical care procedures, such as central line placement, bronchoscopy, endotracheal intubation, and to lead a cardiac arrest (code) team. Site leaders should ensure that critical care resources are present to perform procedures and respond to such emergencies.

## Section 5: Roles and Responsibilities

### Question 5.1: What are the Roles and Responsibilities of the Team Providing TeleNCC?

#### Recommendation 5.1:

The composition of the team providing TeleNCC services will vary according to the level of services required and the TeleNCC model that is adopted—at a minimum, it is a solo NCC physician, and at the other end of the spectrum, the team may comprise a combination of healthcare providers including a NCC physician, a critical care RN, a neurocritical care APP, a NCC fellow, and a pharmacist [4, 8, 14].

The roles, responsibilities, and structure of the TeleNCC team will be clearly defined ahead of time between the spoke and hub site. Regardless of their location, the hub personnel will be available for the predetermined periods of time on the basis of the care model.

#### Responsibilities for TeleNCC Provider:

The respective responsibilities of each provider type vary and delegation and sharing of responsibilities are also flexible in accordance with the various permutations of the TeleNCC team composition. Here we recommend the minimum expected roles and responsibilities for the various members of the TeleNCC team with additional suggestions for expanded and optional responsibilities (Table 3) [8].

**Table 3:** Roles and responsibilities of TeleNCC providers and collaborative staff at the hub and spoke sites

	Hub NCC physician	Hub critical care RN (optional)	Hub or spoke NCC App (optional)	Hub NCC fellow (optional)	Hub NCC pharmacist (optional)	Spoke physician/nonNCC App	Spoke critical care RN
*Education/training*							
NCC certification	R	N/A	V	N/A	V	N/A	N/A
Knowledge and training in NCC and critical care concepts	R	R	R	R	R	V	R
ENLS certification	R	R	V	R	V	V	V
NIHSS certification	R	R	R	R	N/A	V	R
Brain death certification	V	N/A	V	V	N/A	V	N/A
EEG training	R	N/A	V	V	N/A	N/A	N/A
TCD training	V	N/A	N/A	V	N/A	N/A	N/A
Telemedicine training	R	R	R	R	R	R	R
Primary (in-person) provider assigned	N/A	N/A	N/A	N/A	N/A	R	N/A
*Clinical responsibilities*							
Available on-site to respond to emergencies	N/A	N/A	N/A or R	N/A	N/A	R	R
Bedside care	N/A	N/A	N/A or R	N/A	N/A	R	R
Available for TeleNCC consultations	R	R	R	R	R	N/A	N/A
Initiate TeleNCC consults	N/A	V	V	N/A	N/A	R	V
Triage	V	V	R	V	N/A	N/A	N/A
Coordination of patient flow between hub and spoke	N/A	V	R	R	N/A	R	R
History/exam	R	N/A	R	R	N/A	R	N/A
Participate in rounds	R	R	R	R	V^1^	R	R
Respond to NCC emergencies via telemedicine	R	V	R	R	V	N/A	N/A
Monitoring physiological data and respond	V	N/A	V	V	N/A	R	R
Recognize and interpret physiological data	R	N/A	R	R	N/A	R	R
Interpret EEG/TCD	V	N/A	V	V	N/A	N/A	N/A
Developing Treatment plan	R	N/A	R	R	V	R	N/A
Anticipatory guidance	R	R	R	R	V	N/A	N/A
Communication between hub and spoke	R	R	R	R	R	R	R
Execute treatment plan	N/A	N/A	N/A	N/A	N/A	R	R
Family communication	R	N/A	R	R	N/A	R	R
EHR documentation	R	V	R	R	V	R	R
Systematic handoffs	R	R	R	R	N/A	R	R
Brain death exam	V	N/A	V	V	N/A	R	N/A
Med review/reconciliation	N/A	N/A	N/A	N/A	R^2^	N/A	N/A
*Quality and research*							
Education (e.g. debrief, just-in-time, lectures, conferences)	V	V	V	V	V	R	V
Peer review/RCA/QI	V	V	V	V	V	R	R
Staff meetings	V	V	V	V	V	R	R
Trial enrollment	V	N/A	V	V	V	V	N/A

Those which are recommended as required are represented by “R,”, whereas those that are variable or voluntary are represented by “V”

^1^The HUB or SPOKE pharmacist should be available for daily rounds, prospective patient chart review to facilitate recommendations, and ad hoc consultations

^2^The HUB or SPOKE pharmacist is required to review and reconcile medications

#### Hub Teleneurointensivists:

This will be an intensivist who is board-certified or board-eligible in neurocritical care [14]. We recommend in all cases this is a telemedicine neurocritical care expert consultant advising the primary in-person admitting physician (or clinical provider) of record for the patient(s); they additionally require an in-person spoke site proceduralist partner.

At a minimum, this hub Teleneurointensivist is responsible for: obtaining history and examination with the assistance of the spoke personnel, viewing of the raw imaging, EEG, transcranial doppler (TCD), etc., and interpretation of the results, creating a treatment plan with the spoke providers and provide anticipatory guidance (e.g., what to do and when to reach back to the hub), and communicating with the patient and their family, including participation in family meetings with the in-person team to discuss neuroprognosis or goals of care. It is recommended for documentation to use a standardized template in EHR and to perform systematic handoffs (*Please refer to Documentation standards in Structure *Sect. 3*, and Activation and Communication section for recommendations on time to activation and triage for emergent consults, *Sect. 6*).*

The optional and expanded responsibilities include the following. First, facilitating and supervising the spoke providers’ brain death exam, but not acting as the legally responsible in-person examiner. Assisting the in-person provider’s performance of a brain death examination can be particularly helpful if the provider has less experience and in circumstances where two brain death exams are mandatory. Next, the TeleNCC provider may perform regular education and mentoring of the spoke team, and host debriefing meetings after critical events. Third, they may also participate in staff meetings, educational conferences, peer review, root cause analysis (RCA), and quality improvement (QI) activities. Finally, they may help enroll for NCC clinical trials/studies in decentralized clinical trial arrangements.

#### Spoke or Hub TeleNCC-Specific Advanced Practice Provider

Requirements for the spoke or hub TeleNCC APP at the minimum are: (1) NIHSS certification, (2) demonstrated training and experience necessary for eventual neurocritical care competency, and (3) appropriate institutional credentialing for practice in neurocritical care.

The provider ideally should have the opportunity to become certified in ENLS and have work experience in a dedicated in-person NCC. This learning experience should be focused on understanding triage and management of the comprehensive ICU neurological examination, management of neuroinvasive monitors and therapies, and neurocritical care emergencies for core disease pathologies. In the most optimal scenario, panelists felt that this training period in an in-person NCC should be at least 1–2 months.

The specific required *minimal responsibilities* are triage, initial response to NCC emergencies, obtaining history and conducting examination independently or via video with the hub provider (if physically present as the spoke APP), participating in rounds, collaborating on treatment plan, communication between spoke and hub teams for execution of plans, provide anticipatory guidance and be available for questions, family communication, documentation using a standardized template in EHR and systematic handoffs.

*Optional and expanded responsibilities* include completion of neurocritical care advanced practitioner fellowship, participating in peer review/RCA, QI and conferences as needed, helping patient enrollment for NCC trials/studies and, in some states, supervising the brain death exam.

#### Hub NCC Fellow

The TeleNCC fellow performs essentially the same functions as the board certified neurointensivist; however, fellows should receive supervision or oversight by neurocritical care faculty in compliance with the ACGME and institutional guidelines. The supervision of the fellow by the TeleNCC physician may be done on site simultaneously or via multi-presence functionality on telemedicine systems.

#### Hub TeleNCC Pharmacist:

*Minimum requirements *include: (1) a doctorate degree in pharmacy, 2) PGY1 residency or 5 years of clinical experience.

*Optimal requirements: *(1) board-certification in critical care pharmacotherapy (BCCCP) (2) have completed a PGY2 in Critical Care Pharmacy, and (3) certification in ENLS.

For the non-TeleNCC pharmacist at the spoke hospital, it is recommended that there is clear communication and roles as to responsibilities for primary medication management, cross-checking, and advanced monitoring when specific neurological medications are used. The pharmacist is typically working asynchronously with the TeleNCC team and is actively discussing cases on a routine daily basis.

*For a full description of pharmacy roles, please refer the Pharmacy *Sect. 10.

#### Spoke Physician and Non-TeleNCC APP:

The spoke site provider staffing will vary depending on the capabilities and resources of the spoke hospital. Some may have intensivists with or without APPs on site and need TeleNCC for consults on an ad hoc basis and for overnight coverage. Other hospitals may have a neurohospitalist or anesthesiologist staffing a smaller ICU and will be looking for dedicated TeleNCC assistance 24/7. The spoke site physician will be the attending of record.

*Required Responsibilities of the Spoke Physician/APP* include initiation of the TeleNCC consult for patients with NCC needs (conditions that require NCC care should ideally be identified in advance on the basis of discussions with spoke/hub), assist with history and remote examination (telepresenter role), family communication, and placing orders. This provider should develop treatment plans with the TeleNCC provider and execute these plans. They must be present on site to monitor patients, inform TeleNCC personnel of any major clinical changes or results, and perform emergent procedures. This clinician should ensure appropriate handoffs including guidance as to when to reach back to hub, adequate EHR documentation, perform brain death exams, ensure safe and smooth transfer of patients to hub, and participate in RCA/peer review, conferences, QI initiatives.

#### Spoke Critical Care TeleNCC Team Lead RN and Non-TeleNCC RN

The TeleNCC Team lead RN is a spoke hospital TeleNCC-focused RN, with specific interest and training in Neurocritical Care. In some institutions, this is referred to as the “Spoke Neuro RN Champion.” This role is not a requirement for the spoke TeleNCC service, but rather an optimal component in the ideal setting.

If there is such a provider fulfilling this role, to meet their responsibility and role requirements, we recommend: (1) the requisite critical care nursing experience and neurocritical care nursing experience, (2) NIHSS certification, and (3) the ability to perform a neurocritical care examination, especially the coma exam. For specific credentialing, the RN should have some sort of specialty neuroscience (e.g., SCRN, NVRN, CNRN) or critical care board certification (e.g., CCRN).

This provider RN is the rounding spoke-partner for the hub provider. They may function as a triage (or first call), be present during rounds and provide nursing support with just-in-time, anticipatory guidance, and regular education to the spoke nurses. They should also be able to actively liaise with any admission and transfer centers or hospital command centers and ensure smooth and safe transfer of patients from spoke hospital with systematic handoffs. Importantly, the RN should ensure recommendations are implemented and carried out.

Optional and expanded responsibilities include: ENLS certification, participating in peer review/RCA, QI, and staff meetings as needed.

The non-TeleNCC RN is an intensive care unit RN who works with the TeleNCC team but usually does not have formal training or experience in TeleNCC nursing. This provider at a minimum should have training in how to perform a coma exam and, optimally, a NIHSS exam.

## Section 6: Activation and Communication

### Question 6.1: What Method of Communication Should Providers Use with TeleNCC?

#### Recommendation 6.1:

The optimal communication processes may vary depending on the needs and local culture of individual TeleNCC networks. A TeleNCC team may carry a dedicated phone or single contact phone number that forwards all calls to the provider on duty, allowing direct access to the consulting neurointensivist. Alternatively, they may be contacted via phone call, text, or paging system or mobile app, through an on-call provider listing, transfer center, or some combination of these.

#### Rationale 6.1:

A direct phone line to the teleneurointensivist may be more efficient than communicating through a transfer center, which would require a third party to connect the two teams. A direct phone line may offer a greater sense of support to the consulting providers, reduce communication barriers, and build a trusting relationship. If direct contact through a phone is used, teams will need to specify whether communication must be done only via phone call, or whether texting is also acceptable. If so, they must ensure that a secure method is used to protect private health information [4].

Alternatively, making connections through a transfer center may provide other advantages. When activating a new TeleNCC consult, a transfer center representative may be able to gather the most important information to present to the teleneurointensivist (e.g., patient identifier information, reason for consult, consulting team contact information, urgency of consult). If the teleneurointensivist is unavailable at the time of a new consult request, a transfer center representative may be able to triage the urgency of the call and escalate calls to a backup neurointensivist if needed. A transfer center may also increase efficiency by attempting to contact a busy outside hospital provider directly and then connecting the TeleNCC provider when they are available. If a patient ultimately does require transfer to a higher level of care, the transfer center team will already be aware of the patient and can start making the necessary arrangements. Utilizing a transfer center may also be helpful for efficiently tracking data [8].

### Question 6.2: What Processes Should be Established Regarding Activation of TeleNCC Consults?

#### Recommendation 6.2:

The panelists recommend that activation of a consult should be via pager or phone with response time to triage the consult in less than 10–15 min. The time to initiate the actual consult request will vary depending on the urgency of the initial request, and initial triage conversations are suggested to prioritize multiple consults.

It is important to establish clear expectations regarding initial communication between the teleneurointensivist consultant and in-person primary teams, including what information is needed when activating a consult, who activates the consult, which types are covered between TeleNCC and telestroke or teleneurohospitalist services (if available), and how quickly a response from the TeleNCC team should be expected.

#### Rationale 6.2:

The TeleNCC team should identify the type(s) of providers that are optimally making the consult and also the information needed for an initial consult request, such as: patient identifiers, reason for consult, urgency of consult, and in-person primary team contact information. If a consult is urgently needed, this should be indicated, to allow for appropriate triaging of calls.

We recommend an expectation of connecting over the phone within 10–15 min of an initial request for consult. However, if the urgency is made clear at time of initial request, then a shorter time response should be expected. Early connection with the consulting provider allows the teleneurointensivist to triage the urgency with which the actual video consultation should be performed. Preferred times to be called for general questions versus emergencies should also be established at the outset.

If the initial consult request is communicated via a page or a text it, can allow the teleneurointensivist an opportunity to prioritize the urgency with which consults are managed (vs. a direct call to a phone which cannot be triaged). When an urgent request is unable to be addressed quickly, there should be a plan for escalation to a backup teleneurointensivist [13]. The panelists felt that it was not relevant to parallel the same times expected for stroke alert responses, given the nature of neurocritical care problems, especially as there are gray zones between routine versus emergent consults. Time to respond may also vary depending on the system, and the structural components of how response to “neurological emergency codes” are organized at the spoke.

### Question 6.3: What Does the TeleNCC Consultant Need to Communicate Back to the Spoke Team Regarding the Initial Consult, and How?

#### Recommendation 6.3:

Once the two-way audio–visual TeleNCC evaluation has been conducted, clear recommendations should be communicated to the spoke hospital consulting team, both verbally and via documentation in a summary consultative note contained in the EHR.

#### Rationale 6.3:

Recommendations should be presented directly over the telemedicine camera if the primary spoke provider is present in the room during the evaluation, from the on-site spoke TeleNCC PA or NP (if an advanced practice provider (APP) rounded with the hub TeleNCC provider), or via a follow up phone call. In addition, recommendations should be documented in a permanent consult note which is maintained as part of the EHR. This may be entered directly into the spoke EHR or entered into the TeleNCC’s EHR and transmitted to the requesting hospital for uploading.

Furthermore, the TeleNCC team should communicate whether it plans to continue to follow the patient or to sign off. The TeleNCC provider should also specify the preferred method for the in-person team to reach out for additional questions. If follow up is needed, the TeleNCC consultant should establish whether rounding is planned for a specific time on the next day or whether the consultant will reach out to a member of the spoke team in advance to round on the patient [8].

*Please see the Sects.* 7 *and* 3 *on: Afterhours and Anticipatory Guidance and Structural Elements for additional recommendations of content to be included in the consult note.*

### Question 6.4: What Ongoing and Follow-Up Communication is Needed Between Teams After the Initial Consultation is Completed?

#### Recommendation 6.4:

It is important to maintain clear communication channels between spoke teams and TeleNCC, and also between TeleNCC providers. When follow up questions arise, outside of regularly planned rounding times, it is important to establish if and how TeleNCC teams can be reached. The primary spoke medical provider should remain first call and contact (alternatives include TeleNCC APP or RN) and then the TeleNCC team.

#### Rationale 6.4:

Spoke hospital teams should determine which personnel should reach out to the TeleNCC consultant for initial consultations and follow-up and to which personnel this responsibility may be delegated in emergencies (primary attending, APPs, nurses, trainees, etc.). Sites with sufficient staffing and volume may establish a small number of TeleNCC “champions” who are trained in neurocritical issues, familiar with the neurologic exam, experienced with telepresenter role, and accustomed to interacting with members of the TeleNCC team. The TeleNCC consultant should indicate for what circumstances or indications the in-person primary team should reach out with questions. This may include changes in the neurologic exam, once specific tests are completed, changes in EEG, or family present with specific neurological questions. The panelists felt that there should be follow-up encounters or check-in on the same day or subsequent day after most, if not all, TeleNCC consults, as usually done with in-person neurocritical care.

Next, communication with family members and other surrogate decision makers should be delineated at each spoke site. Most panelists agreed that when TeleNCC is discussing sensitive topics, poor or unanticipated outcomes, and important goals of care that this should be jointly done with the spoke provider, if possible. However, it was recognized that a balance of efficacy, inclusivity, and efficiency exists with coordinating difficult conversations.

Finally, it is important to maintain clear communication amongst the TeleNCC providers themselves. This includes maintaining an updated on-duty coverage schedule and a system for signing out to one another between shifts. It should be established whether this is done verbally, written in the EHR, over email, or other secure mechanisms for protected health information [4].

## Section 7: Afterhours and Anticipatory Guidance

### Question 7.1: What Should the Availability of TeleNCC be for Utilizing Centers?

#### Recommendation 7.1:

TeleNCC programs should be prepared to provide access 24 h a day, 7 days a week, 365 days a year, unless the coverage is off-hours or backup for onsite NCC providers. Programs that are unable to provide continuous 24/7 coverage should ensure that an alternative service with appropriately trained and credentialed neurocritical care providers is available to fill any gaps in coverage. A combination of in-person and TeleNCC coverage is reasonable so long as coverage periods are well defined. Therefore, TeleNCC coverage schedules should be developed far in advance of any anticipated coverage gaps to ensure that utilizing hospitals know who to contact at all times.

#### Rationale 7.1:

Neurologic emergencies can occur at any time and often require rapid assessment and treatment to minimize secondary injury and maximize the potential for good outcome. Noncritical care trained neurology staff at referring hospitals may be unable to accurately assess the acuity of a neurologic syndrome and will likely rely upon the teleneurointensivist to inform the time-sensitivity of recommended steps in the workup and management. While many inpatient telemedicine consult services are based upon a daily virtual rounding system, it is essential that TeleNCC systems also integrate a time-sensitive process for responding to new consults and addressing follow up questions and concerns, including overnight and on weekends and holidays.

### Question 7.2: What Sort of Anticipatory Guidance Should be Provided?

#### Recommendation 7.2:

When providing consultative support, TeleNCC providers should make every effort to include contingencies for an appropriate range of anticipated and unanticipated but possible clinical trajectories. These management recommendations should include the appropriate responses to the spectrum of possible findings on recommended diagnostic studies as well as contingencies for the most likely foreseeable complications that may arise in a given case (e.g., first, second, and additional treatments to be implemented in response to breakthrough seizures on EEG, or a stepwise approach to the management of refractory intracranial hypertension). To the extent possible, these contingency plans should be based on published evidence-based treatment guidelines, in particular those endorsed by all the applicable professional societies. These would ideally be codified through standardized treatment protocols and provided to participating hospitals for easy access outside of the formal consult route as well.

#### Rationale 7.2:

While not every foreseeable contingency can possibly be accounted for, providing referring centers with a detailed management plan that considers the range of potential clinical trajectories yields a multitude of benefits. For the patients, appropriate anticipatory guidance will allow for rapid initiation of indicated treatment. For TeleNCC providers, appropriate anticipatory guidance would ideally minimize the need for multiple follow up calls, streamlining the ability to provide care for multiple patients simultaneously.

### Question 7.3: How to Ensure Engagement with Patients’ Families?

#### Recommendation 7.3:

We suggest that each TeleNCC system establish a system by which patients and their families have access to direct interaction with TeleNCC providers so that they may ask clarifying questions about the patient’s condition, understand the proposed treatment recommendations, and gain insight into the prognosis. There is a specific, distinct benefit of TeleNCC to provide neurological prognosis insights. This added value of care should be considered in each family or surrogate discussion.

#### Rationale 7.3:

In addition to providing acute management recommendations, one of the key values of neurocritical care is being able to provide patients and families with expert information about the anticipated clinical course and longer-term neurologic prognosis. While the structure of family interactions is likely to vary from program to program on the basis of clinical bandwidth and other specifics of the program structure, it is essential that referring centers have a reliable pathway through which patients and their families may connect with the TeleNCC provider [24]. Ideally, these interactions would occur in the context of a dedicated family meeting and would be available within a reasonable timeframe of family request [25]. Recognizing that this may not be feasible in all systems, this may require including families in the daily rounding or check in with the clinical team [26].

## Section 8: Regulatory and Credentialing

Note—this section focuses on USA-based topics and scope of coverage, which is relevant to the non-USA practitioner. The scope of International regulatory landscape is rapidly changing and also too extensive for this publication, hence it is recommended to check locally and regionally for country specifics.

### Question 8.1: To What Degree Should There be Interstate Credentialing Support: Interstate Compact, Full Credential Versus Telemedicine Only?

#### Recommendation 8.1:

Measures to reduce the administrative burden of obtaining incremental licenses should be fully supported, including the adoption and diffusion of licensing compacts, where not yet been enacted. As an additional measure, state jurisdictions are encouraged to promulgate telehealth registration processes to further expedite out-of-state clinicians obtaining authorization to practice telehealth, while still ensuring the oversight of the receiving state’s licensing board.

#### Rationale 8.1:

Pursuing multiple licenses can be extremely taxing for clinicians—and once obtained, maintaining multiple licenses imposes challenges and administrative burdens. As technology advances to enable the provision of high-quality care and the transfer of professional knowledge across distances, the regulation of professional practice should similarly advance without imposing added barriers to care. Where possible, measures to minimize administrative burden should be encouraged, including streamlining of licensure process(es) and standardization of CME requirements between states.

### Question 8.2: How Should Hospital Credentialing and Privileging Measures (Independent vs. Credentialing by Proxy (CBP)) be Structured?

#### Recommendation 8.2:

It is recommended that CBP arrangements, which allow the spoke hospital to recognize the credentialing measures by the hub hospital, be pursued whenever amenable to the spoke site. The spoke site should retain the ability, contractually, to trigger full credentialing measures on an as-needed basis or subject to specific conditions.

#### Rationale 8.2:

Traditional credentialing measures are time-consuming and costly. While these credentialing requirements play an important role in protecting patient safety by vetting the expertise and qualifications of the healthcare workforce, the current requirements are more than sufficient to meet this goal. The spoke and hub sites for a telehealth interaction are each independently subject to the same Conditions of Participation (COP) requirements and consequently the same standards imposed by their applicable accrediting body. It is duplicative for the hub site to thoroughly vet the expertise of (e.g.) a TeleNCC provider only to have the expertise revalidated by the site. The hub site subsequently should also have the obligation to report any deficiencies with state or federal state standards later discovered with its own credentialing measures and mitigate deficiencies to the satisfaction of any spoke sites that have relied on those measures.

### Question 8.3: Should the TeleNCC Consultant be Credentialed for the Specific Hospital, and Thereby Able to Act/View the Local Spoke Site’s EHR?

#### Recommendation 8.3:

The TeleNCC consultant should be credentialed by-proxy (CBP) to the spoke site’s hospital. The spoke site should define the appropriate scope of access for providers credentialed under CBP measures.

There should be a minimal, standardized amount of required training for spoke EHR access, as the provider both abides by HIPAA regulations and should be compliant with local security and EHR usage training at their hub institution. Each spoke site, bound by their given accrediting body standards, will need to demonstrate the completed training upon survey request. However, it should be the hub site’s responsibility to complete full verification of credentials and training and report to the spoke sites accordingly. It would be reasonable to provide a simple 1-page standardized format document for TeleNCC provider to sign that is an agreement to adhere to proper use policies and procedures of the spoke EHR and its data.

#### Rationale 8.3:

CBP has several efficiencies for providers working via telemedicine, and current regulations require sufficient onus on the part of the hub site to evaluate the education, expertise, and competency of a provider. Given the industry’s move toward (1) health systems and (2) integrated medical records, a spoke site may be part of larger system with multiple sites; meaning that, a TeleNCC provider could potentially be given broad access to view, use, and document in that spoke site’s medical record in conducting the work. Unchecked, this could lead to patient privacy concerns or other concerns with the integrity of patients’ health information. As CBP measures are implemented, this does not alter or remove the spoke site’s concurrent obligations to implement appropriate technical safeguards to protect health information. As each healthcare organization may design its EHR differently, even across the same vendor, it is difficult to draw generalizations for appropriate access. Instead, our recommendation is for governance of each organization to articulate the appropriate access for any provider credentialed under CBP measures, any relevant policies to direct the actions of telehealth workforce, as well as the cadence under which technical permissions are reviewed.

### Question 8.4: What Other Risk Mitigation Measures Should be Observed in Contracting for Virtual Consultations Between Healthcare Facilities?

#### Recommendation 8.4:

Prior to the implementation of TeleNCC consultations, contractual agreements must be in place to define the scope of and liability for the service to be performed.

The agreement should define duration and term of the arrangement, applicable statutory and regulatory requirements, and most importantly the expected obligations and scopes of work for each party to the agreement. It is advised that the parties define the expected organization’s obligations with specificity, including billing responsibilities, recordkeeping requirements, rights of access to records, and similar processes related to complying with applicable law. The agreement may also include protocols for participation quality improvement and after-action review(s), on an as-needed basis if desired by the parties. The liability and malpractice coverage should be defined in the initial and annual contracting and risk assessment process, which is case by case and unique to each hospitals’ and health systems’ arrangement. It is recommended that each hub site that carries the primary contracting for the provider pay close attention to and review of all the liability agreements prior to engaging in TeleNCC.

#### Rationale 8.4:

Organizations are obligated to maintain and retain inter-company agreements defining the scope of external services involved in their operations. These are frequently outlined and reviewed as a part of Joint Commission (or other accrediting body) survey activity. It is recommended that organizations approach the contracting phase with discernment, to ensure that once the service comes to fruition each party knows its’ expected obligations to comply under the arrangement.

#### Extended Rationale of State Licensure and Credentialing Concepts

*Licensure, Professional Scope of Practice, and Pathways to Cross-State Licensure*

The licensing of healthcare professionals falls within the regulatory authority of each state. Licensing Board structures may vary from state to state. However, each type of licensed health profession, generally, has its own licensing board charged with overseeing the standard of care, the appropriate scope of practice, and disciplinary consequences of the individual licensees under the board’s purview [27].

The provision of video-based telemedicine consultative care is ubiquitously agreed upon by state boards of medicine as a type of clinical interaction which would constitute the practice of medicine or the healing arts; therefore, would fall under the regulatory enforcement of the corresponding state board and require a license for a healthcare professional to engage in that activity within that state [28]. Professionals must, therefore, comply with the relevant standard of care owed to the patient, reiterating the importance of clearly articulated care protocols and patient eligibility criteria upfront to match patient needs with the right level of care.

The provision of clinical care across state lines increases licensure complexity. Providers caring for patients via video will need licenses in both the state where they are located and where the patient is located at the time of the service. In addition, providers must adhere to the applicable scopes of practice standards for both states.

Multiple types of legislative vehicles exist to increase licensure portability across state lines and can reduce some administrative burden to providers when pursuing new licenses. Licensure compacts provide healthcare professionals with an expedited path to a new license in another state that has also enacted the compact. Multiple states have enacted *the Interstate Medical Licensure Compact for Physicians* in recent years [29]. The IMLC is not a reciprocal license structure, i.e. a compact that grants authorization to practice in the new state, rather is an expedited avenue to a new license issued by a new medical board, and thus still entail separate fees and education credits, among other requirements [30].

Separately from licensure compacts, some states have enacted telehealth registration pathways or “special purpose licenses” which allow out-of-state providers to register with the local medical or nursing board to see patients within that state via telemedicine. Where available, these pathways can be efficient means of gaining authorization to practice as an out-of-state provider (usually for a lesser fee than a full or compact license)—though, as of this writing, these types of registration structures are far from the norm across the nation.

#### Credentialing Requirements and Models

Provision of video-based telehealth services also implicates site-based credentialing requirements. Credentialing measures, generally, serve a critical patient-safety function, ensuring that a healthcare organization verify the education, professional credentials, and competence of each provider performing services under its organization per the governing standards delineated in its organizational bylaws. These internal credentialing obligations also trigger external accountabilities. CMS’ Conditions of Participation (COP) for the Medicare program impose standards for on-site credentialing. In addition, a healthcare organization may be subject to survey on these credentialing measures through The Joint Commission (TJC) or other accrediting body [31].

As technology evolved, allowing for the diffusion of medical expertise via video, CMS introduced the ability to streamline credentialing requirements for telemedicine through credentialing by proxy (CBP) arrangements; TJC and other accrediting organizations also observe these processes. CBP arrangements, permit spoke site hospitals to streamline credentialing measures for telemedicine-based services and maintain compliance with their COP requirements. CBP essentially works as follows: by written agreement, the spoke site hospital agrees to recognize the recognize the credentialing measures undertaken by the hub site hospital or telemedicine entity (i.e., where the consulting provider is located) [32].

## Section 9: Finance

Most recommendations are written within the USA health insurance framework, which may be applicable to some but not all institutions within and outside of the USA Guidelines and telehealth policy are changing rapidly; this section provides additional references and resources to assist organizations to evaluate and establish a telehealth program [33–35]. We note that financial arrangement and contracts undergo continuous change; hence, it is worthwhile to concurrently compare these recommendations with others’ within a similar scope that may be more recent (Telestroke, Telecritical Care).

### Question 9.1: What are the Optimal Financial Options for Funding and Reimbursement of TeleNCC Services?

#### Recommendation 9.1:

**Hub:**

For TeleNCC video services, the billing type will vary on the basis of the organization of the health system and the associated contractual agreements. In most cases, the provider should bill a telemedicine specific code, detailed below. Alternatively, the provider may not directly bill themselves per encounter when the contract is based on per click consultation rates, with or without a base contractual reimbursement.

In some circumstances, when possible, the provider may bill as the In-Person Equivalent CPT code as if the patient was seen in a healthcare facility (i.e. Facility to Facility). If the hub provider is performing a Critical Care visit to a critically ill patient and is acting as the managing clinician, then the provider should bill the Critical Care CPT codes: 99,291 and intervals of 99,292 (Table 4). If the provider is providing expertise only acting as a consulting clinician, for either a critically ill or noncritically ill patient, then the CPT G codes may be used.

**Table 4:** Billing codes used in TeleNCC based on type, severity, and role of the clinician

	CPT	Time requirement (min)	Initial or follow-up (subsequent)	Telehealth or in-person	Audio only allowed	Setting	Level of care	Care requirements (expertise or managing)	Provisional or permanent
Critical Care and managing physician	99,291	First 30–103	Initial	In-Person equivalent	No	Inpatient	Critical	Managing clinician	Provisional
	99,292	Additional 30 min increments (× 1, × 2, × 3, × 4)	Follow-up	In-Person equivalent	No	Inpatient	Critical	Managing clinician	Provisional
Critical or non-critical care and providing expertise	G0425	30	Initial	Telehealth	Yes	Inpatient or emergency	Critical or non-critical	Expertise (consult)	Permanent
	G0426	50	Initial	Telehealth	Yes	Inpatient or emergency	Critical or non-critical	Expertise (consult)	Permanent
	G0427	70	Initial	Telehealth	Yes	Inpatient or emergency	Critical or non-critical	Expertise (consult)	Permanent
	G0406	15	Follow-up	Telehealth	Yes	Inpatient or emergency	Critical or non-critical	Expertise (consult)	Permanent
	G0407	25	Follow-up	Telehealth	Yes	Inpatient or emergency	Critical or non-critical	Expertise (consult)	Permanent
	G0408	35	Follow-up	Telehealth	Yes	Inpatient or emergency	Critical or non-critical	Expertise (consult)	Permanent
Non-critical care and providing expertise	99,221	40	Initial	Telehealth	No	Inpatient	Hospital (non-critical)	Managing clinician	Provisional
	99,222	55	Initial	Telehealth	No	Inpatient	Hospital (non-critical)	Managing clinician	Provisional
	99,223	75 >	Initial	Telehealth	No	Inpatient	Hospital (non-critical)	Managing clinician	Provisional
	99,231	25	Follow-up	Telehealth	No	Inpatient	Hospital (non-critical)	Managing clinician	Permanent
	99,232	35	Follow-up	Telehealth	No	Inpatient	Hospital (non-critical)	Managing clinician	Permanent
	99,233	50	Follow-up	Telehealth	No	Inpatient	Hospital (non-critical)	Managing clinician	Permanent

*Both MD and APPs are able to bill all in table

Under the current USA regulations for telehealth care, if the patient is noncritically ill and the provider is acting as the managing clinician, then the 99,221–99,233 CPT codes may be used. Note that some CPT categories allow for audio only consultation and all have time-based requirements for the respective initial versus follow-up categories. In these models, the hub site bills a professional fee for the work performed by the physician or qualified healthcare practitioner.

Another option, though often less preferred, is via the Provider-to-Provider asynchronous e-consult whereby only the hub site is reimbursed for their time. The CPT codes for these include: 99,446, 99,447, 99,448, 99,449, 99,451, and 99,452. e-Consults cannot be billed if “Facility to Facility” (above) is billed either 14 days before or after the e-consult. In addition, each CPT code has its own requirements, i.e. 99,446–99,449 conclude with a verbal opinion report and written report. 99,451 requires only a written report. 99,452 is related to when a provider is referring a patient to another provider and is billed if spending 16–30 min in a service day preparing for the referral.

**Spoke:**

For TeleNCC video services, the spoke site may be able to bill a technical facility charge (i.e. Q3014). The availability of this facility fee will vary by payer. For example, CMS does pay a facility fee under Part B of the Medicare program, while not all commercial and state-level medical assistance plans recognize or reimburse for this component of the telehealth service.

#### Rationale 9.1:

The above recommendations assume that the virtual services are being billed to third-party payers. Contractual arrangements between separate entities may have different facets, including subscription-based models for professional expertise for the distant site provider.

Panelists suggest consideration of Alternative Payment Models or Value-Based Payment arrangements that reward either total cost of care (TCOC) accountability or condition/episodic model. Total cost of care refers to a patient’s entire cost of medical care and expenses within a defined period. Generally, in value-based arrangements, if the system spends less than the TCOC target for a certain patient population, there is opportunity to share in savings and garner additional revenue.

The panelists recommendations are intentionally broad and nonprescriptive as to be applicable across multiple systems and within the context of the current fee-for-service (FFS) of the U.S. Health Care system. They are intended to be implemented in the context and regulations of existing billing requirements, including anti-kickback policies. We caution that the recommendations for individual institutional risk tolerance assessment and understand that new guidelines and reimbursement policy are changing rapidly and will need to be updated by 31 December 2024 and thereafter.

### Question 9.2: Which Variables Should be Captured in the Fee for Service or System-Based Fee of TeleNCC?

#### Recommendation 9.2:

Given the limited TeleNCC financial impact assessment and efficacy data, it is warranted to have individual healthcare systems considering TeleNCC to perform a cost-effectiveness analysis, cost-utility analysis, and/or healthcare utilization analysis to determine the Return on Investment (ROI) / Net Operating Income (NOI) modeled over multiple years.

## Sub-Questions :

### What are Operational Cost Considerations and Estimates to Provide TeleNCC?

#### Recommendation 9.2A:

Costs to implement tele-critical care programs vary widely but generally should consider the following categories for financial impact assessment: technology, staffing, real estate, and hospital variables.

#### Rationale 9.2A:

In the current economic landscape, it is a challenging business proposition to have hospital systems invest in TeleNCC that requires additional upfront revenue and should evaluate financial impact. Careful consideration for variables in cost return analysis should be done, as follows. Technology includes hardware, software, networking services, technical support, and communication fees. Staffing includes training, salaries, support services, as well as elimination of spoke staff who would be theoretically underutilized due to low volumes. Hospital variables are multiple and include the pharmacy, laboratory, radiology, ancillary support and procedural services [36]. An opportunity evaluation of TeleNCC to allow the hospital to upgrade its comprehensive stroke and/or trauma care may offset startup and investment costs.

## Subquestion 9.2B:

### What is the Evidence and Calculations for System-Based Cost Impact or Savings for TeleNCC?

#### Recommendation 9.2B:

Current evidence regarding the generalized use of telehealth, including TeleNCC specifically, and its correlation with utilization and cost-reduction is not conclusive. Practices should consider evaluating cost-savings, cost-reduction, utilization, Quality Adjusted Life Years (QALYs), transfer rates, bed availability, and salary and benefits.

#### Rationale 9.2B:

In the current economic landscape, it is important to better understand the costs and effect to determine health system investment in TeleNCC. Approaches for evaluating cost-savings include the impact of shared staffing model, specialty specific evidence such as patient demographics and outcomes (i.e. LOS, age, stroke severity), QALYs, transfer rates and method of transfer (i.e. helicopter), and indirect impact on bed capacity.

Current Medicare provisions allow billing for TeleNCC services through the use of in-person equivalent, time-based CPT coding. Hospital systems should consider evaluating the financial impact of investing in TeleNCC given that it requires additional upfront revenue. Should permanent billing codes be created, the price for the services provided should take into account, and be aligned with, the comprehensive cost and resources to provide the service including implementation and maintenance of the service, volume of patients served, equipment, provider time, data costs, IT costs, security requirements, system integration, and resource intensiveness.

## Section 10: Pharmacist Clinical Services

### Question 10.1: How Should Pharmacists Participate in the Teleneurocritical Care Team?

#### Recommendations 10.1A for Minimum Requirements:

Clinical pharmacist(s) are available for, or included in, spoke multidisciplinary or hub teleneurocritical care team rounds. This can be conducted in-person or virtually, via telemedicine remote review and management. A standard means and time of communication should be established to ensure consistency and limit variation.

Clinical pharmacists, ad hoc, respond to drug information questions, consults, and review medication orders as able by law. An activation and communication mechanism should exist to request a virtual consult when the pharmacist is not in-person at the spoke or hub site.

- Clinical pharmacists should review and provide recommendations for the development of guidelines, protocols, policies, and electronic order sets regarding pharmacotherapy.
- Clinical pharmacists should provide ad hoc education regarding neurocritical care pharmacotherapy to multidisciplinary staff.

Clinical pharmacists collaborate with local, spoke pharmacy services to create a clinically reasonable medication interchange list for when first-line medications are not immediately available at spoke facilities.

Additional details and summary in Table 5.

**Table:** Recommendations for pharmacists and pharmacy services in teleneurocritical care

TeleNCC pharmacist or pharmacy service component recommendations	Minimum	Optimal
Clinical pharmacist available for TeleNCC patient medication review and recommendations	X	X
Pharmacist partial FTE dedicated to TeleNCC team/unit	–	X
Pharmacist with critical care residency or equivalent training	–	X
Pharmacist with expertise in NCC and telehealth	–	X
Discusses patient-specific interventions with team, even if asynchronous	a*	X
Engage in synchronous clinical rounding with team, daily medication review	–	X
Respond to drug information questions and consults	X	X
Available 24 h daily for hyperacute medication review and recommendations (e.g., stroke, status epilepticus)	–	X
Available to review guidelines, protocols, policies relevant to TeleNCC	a	X
Develop guidelines, protocols, policies for pharmacy services in TeleNCC	–	X
Provide neuropharmacology education to multidisciplinary staff	a	X
Participate in quality improvement projects regarding TeleNCC	–	X
Participate in research regarding TeleNCC	–	X
Pharmacy services—24 h day for medication orders/requests	X	X
Pharmacy services—bedside urgent drug list and delivery	X	X
Pharmacy services—policies for appropriate drug review, administration	X	X

Adapted from^14^

^*^a = ad hoc provision of service component

#### Recommendations 10.1B for Optimal Requirements:

The clinical pharmacist working with the TeleNCC team should be a dedicated neurocritical care pharmacist, with critical care specialist training, and ideally board-certification in critical care pharmacotherapy (BCCCP) and should be available either in-person or virtually.

A critical care pharmacist is available 24-h a day to provide TeleNCC collaborative services and ad hoc hyperacute consultation. It is reasonable for the arrangement of each hospital system between the hub TeleNCC providers and spoke providers to take varied forms, yet optimally the pharmacist(s) would:

- Have dedicated NCC/TeleNCC time in their FTE, and
- Be included in multidisciplinary rounds for TeleNCC patients
- It is recognized that conducting a two-way audio–visual encounter with three parties (if two are virtual, i.e., the TeleNCC clinician and the TeleNCC pharmacist) may be technically challenging, hence separate virtual rounds and/or record review and recommendations communicated may be a suitable alternative.

An established division of pharmacy coverage scope and times should be developed and agreed upon by each local spoke pharmacy team and the virtual, or hub, pharmacy team. Irrespective of the location of the pharmacists, we suggest that:

- Critical care clinical pharmacists proactively review neurocritically ill patients to optimize medications and communicate those recommendations in real-time to the TeleNCC team
- Critical care clinical pharmacists help identify drug availability, interchange, and acquisition at facilities.

Critical care clinical pharmacists actively develop, implement, and evaluate TeleNCC guidelines, protocols, policies, and electronic order sets. Critical care clinical pharmacists participate in quality improvement and research regarding teleneurocritical care.

#### Rationale 10.1:

Pharmacists are essential members of critical care teams and their inclusion as part of the multidisciplinary team is endorsed by the Neurocritical Care Society and the Society of Critical Care Medicine [14, 37]. Pharmacists have shown to have a substantial impact on critically ill patient outcomes by decreasing mortality, morbidity, length of stay, length of ventilator days, thrombotic events, and costs, among others [38–40].

There is less research done in telecritical care, however. To date, the implementation of a telecritical care pharmacist has been shown to decrease healthcare costs and improve medication optimization [41]. Many of the impactful interventions pharmacists provide in the intensive care unit (ICU) can be provided in a telehealth ICU, and the inclusion of a critical care trained pharmacist in a teleneurocritical care service brings specialized knowledge and expertise. The role of teleneurocritical care pharmacists, whether in-person or virtually, synchronous or asynchronous, is multifaceted.

- Proactive patient and medication monitoring and optimization: neurocritical care trained pharmacists possess an in-depth understanding of medications used in neurocritical care. Telecritical care pharmacists have optimized medication regimens when proactively evaluating patients including, but not limited to, glucose management, stress ulcer and venous thromboembolic prophylaxis, antithrombotic, antimicrobials, electrolyte management, and analgosedation [42, 43].
- Multidisciplinary virtual rounding: including pharmacist on multidisciplinary rounds creates an opportunity to optimize medications proactively and synchronously. Multidisciplinary rounds including a pharmacist have been shown to reduce the odds of death in critically ill patients [44].
- Consults and therapeutic drug monitoring: neurocritical care patients require close monitoring of their medication response, especially when receiving drugs with narrow therapeutic indices or those requiring titration. Drug consultations can include antimicrobial pharmacokinetic evaluations (e.g., vancomycin), renal dose adjustments, anticoagulation, analgosedation, and others. Specific to the neurocritical care population, pharmacists can play a critical role in therapeutic drug monitoring of antiseizure medications, antimicrobials, and prophylaxis [45].
- Medication reconciliation: neurocritical care patients often have complex medication regimens owing to their acute neurological conditions. Pharmacists led medication reconciliation has led to decreased medication discrepancies, healthcare utilization, and adverse drug events [46]. Medication reconciliation and opiate medication prescribing review could be applied to teleneurocritical services with the goal to mimic services in-person and improve patient care.
- Transitions of Care: critical care pharmacists can contribute to smoother transitions of care, both within the telehealth setting and between different care environments. Pharmacists have decreased adverse drug event-related hospital revisits, emergency department visits, and hospital readmissions by completing medication reconciliation on transitions of care [47].
- Cost-savings/avoidance: telecritical care pharmacists have provided net cost-savings ranging from $237,600 to $489,100 annually [43, 48].
- Quality Improvement and Protocol/Policy Development: critical care pharmacists play a vital role in medication safety initiatives and quality improvement efforts. They can contribute to the development and implementation of protocols, guidelines, and policies related to medication use in neuro critical care. This includes medication protocols for conditions including status epilepticus, subarachnoid hemorrhage, or traumatic brain injury. Their expertise in pharmacotherapy helps ensure standardized and evidence-based practices.
- Research, scholarship, and education: pharmacist can also play an active role in scholarship and research within teleneurocritical to continue expanding and improving such services, evaluate and optimize patient outcomes, and implement and broaden new services [37].

It is important to note that the specific impact on patient outcomes may vary on the basis of the telecritical care program’s structure, the patient population, and the scope of the pharmacist’s responsibilities. However, the expertise of critical care pharmacists in medication management and their ability to collaborate with the healthcare team can generally contribute to improved patient outcomes and reduced costs in telecritical care or TeleNCC settings.

### Question 10.2: How Should the Operational Components of a Pharmacy Department Partner with the Teleneurocritical Care Team?

#### Recommendation 10.2:

Pharmacy services at the spoke facility must ensure the safe procurement, compounding, delivery, and administration of all medications.

Medications commonly used in neurocritically ill patients must be available 24 h daily and administered to the patient within a reasonable time frame, depending on patient-specific factors including disease state and acuity.

When first-line medications are not available at the spoke facility, reasonable alternatives have been prospectively identified and decided upon and are available for timely administration (e.g., if fosphenytoin is not available for a patient with status epilepticus, then IV phenytoin is available, and the compounding and administration is standardized to ensure safety).

#### Rationale 10.2:

Separate, and congruent with the critical care trained pharmacist, the inpatient hospital pharmacy plays a vital role in various aspects of drug procurement, dispensing, administration, and patient outcomes. Here’s an outline of their importance, which ultimately should ensure appropriate drug inventory, delivery, and policies:

1. Drug Procurement: inpatient hospital pharmacies are responsible for sourcing and procuring medications necessary for patient care. They collaborate with suppliers, negotiate contracts, and ensure a reliable supply of medications. This role is crucial in maintaining an adequate inventory and ensuring that the right medications are available when needed, especially when care is administered at smaller, community, or critical access hospitals. The inpatient pharmacy should work with the TeleNCC clinical pharmacist and TeleNCC team to have appropriate alternative medications, when first-line medications are not available.
2. Dispensing Medications: inpatient pharmacies are responsible for verifying provider orders and accurately dispensing medications based on prescriptions/orders by providers. They ensure the right dosage, proper labeling, and appropriate packaging of medications to avoid errors or patient harm. This step is crucial for patient safety and optimal treatment outcomes.
3. Medication Administration Support: inpatient pharmacies support healthcare professionals in administering medications to patients. They prepare specialized medications, such as intravenous (IV) solutions or sterile compounding, ensuring the correct formulation and dosage. By providing these specialized medications, the pharmacy helps ensure patient safety during administration. Furthermore, policies are in place to assist in educating and standardizing the administration of medications.

## Section 11: Quality

### Question 11.1: What Quality Metrics and Review Processes are Important for the Daily Operations of TeleNCC?

#### Recommendation 11.1:

In addition to specific metrics of telecommunication integrity and quality, TeleNCC systems should capture consult volume with time per encounter and characterize the similar quality metrics of daily operations as compared with an in-person NCCU. The panel recognized that it may not be possible for some spoke sites to quantify comprehensive quality metrics; however, there should be a minimum effort made of select metrics. At a minimum, quantification of (a) adherence to previously published standard care metrics for core NCC practice of stroke and seizure care, as well as appropriate disease-specific medication initiation and (b) adverse events should be performed. TeleNCC specifics include time to consult initiation, consult duration, audio-visual failure rates, diagnostic and therapeutic changes, as well as avoidance of unnecessary tests treatments, or transfers (Table 6).

**Table 6:** Examples of measurable quality and outcome metrics for a TeleNCC system. Some items may be disease specific. Telestroke specific metrics are not included in this table

Operational	Structural and technical	TeleNCC specific	Disease specific
Impact on cost and value to health system	Time from call to consult, consultation duration, failure rate	Perceived impact on both hub and spoke staff's workload	Mortality—ideally separate withdrawal of life sustaining therapies versus unanticipated deaths
Transfer rates (spoke to hub, outside facility to spoke and/or hub), avoidance of unnecessary testing or costs	Frequency of new diagnoses given by TeleNCC	Consult volume and time in individual encounters (by tele platform or other means)	Hospital-acquired Infections, deep venous thrombosis rate, duration of mechanical ventilation
Change in volume of care of patients with complex neurocritical care disease	ICU readmission rate	Length of stay (LOS)—hospital and ICU	Adherence to disease specific guideline management, and timely medication administration
Provider satisfaction		Utilization rates of major tests and imaging	Discharge disposition
		Patient satisfaction	Functional outcomes—mRS at discharge, and 90 or 180 days
			NIHSS Score at discharge, and at 90 or 180 days
			Changes in goals of care
			Changes in code status

For both daily clinical operations and outcomes, TeleNCC shall have a systematic quality improvement and performance management process that complies with all organizational, regulatory, or accrediting requirements. Value is best achieved by high quality, easy accessibility, while cost is concurrently controlled. The panelists suggest that a quality and implementation science approach be used.

Note, general critical care quality metrics as recommended by hospital accreditation organizations, as well as existing critical care guidelines, should be upheld. The recommendations here are additive, with focus on the TeleNCC interaction and disease processes.

#### Rationale 11.1:

Quality indicators shall include the administrative, technical and clinical components for the daily provision of TeleNCC services (Table 6). The panel agreed that a TeleNCC service should adhere to similar in-person quality metrics, since the fundamental purpose of TeleNCC is to provide the same level and standard of care as in-person neurocritical care, as described previously [14]. Additional specific measures may be sought after, such as appropriate antimicrobials for meningitis, tiered management of status epilepticus, EVD infection rate, etc. Finally, it is important to note that previously described levels of NCCU care components may not be applicable to all systems, primarily on the basis of the spoke site resources.

Specific process measurements to telemedicine should be continuously or semi-continuously captured, and these focus on the integrity and functionality of the teleportal system. These include: the time from call to consult, duration of consultation, audio–visual system quality, EHR integration, ability to access lab/radiology results, and radiology study readability. These metrics should be documented and reported [13]. Keeping track of adverse events is equally crucial, *as described in Outcomes *Sect. 12.

In the optimal scenarios, it is useful to capture how TeleNCC monitoring changes in patient examination results or identifying signs of decline can also provide valuable insights. Incorporating local site perceptions through surveys adds a qualitative dimension to the evaluation process, offering perspectives from both patients and providers.

The panel agreed that the TeleNCC system leadership should ensure all appropriate systems are in-line with operational standards. This includes system orientation and training, contracts, licensure and credentialing are current, coding and billing for services are accurate, and regular quality reviews are occurring. In addition, panelists had consensus that review of the data and results should be used to improve quality by regular feedback and processes improvement cycles.

Quality outcomes should be used to make technical, programmatic, and clinical changes. These are based upon the best new evolving technology, practice principles, evidence-based guidelines, and clinical research or changing service requirements. TeleNCC staff, administrators, and ICU healthcare professionals should be in alignment to meet specific program outcomes and process measures recognizing that in addition to shared goals, the ICU and TeleNCC might have unique metrics based on their different contributions to the program [2, 13]. A process for the reporting and dissemination of quality metrics and outcomes should be defined for both administrative and operational staff analysis.

## Section 12: Outcomes

### Question 12.1: What Outcome Metrics Should be Captured to Measure the Overall Effectiveness of TeleNCC?

#### Recommendation 12.1:

The panel recommends the following outcomes are measured: transfer rate from the spoke to the hub, adverse events (examples below, in rationale), hospital and ICU length of stay (LOS), in-hospital mortality, discharge disposition, and National Institutes of Stroke Scale (NIHSS) and modified Ranking Scale (mRS) at discharge. At a minimum, the transfer rate from spoke to the hub site, mortality, and discharge metrics of NIHSS when appropriate and mRS should be compared with in-person NCCUs for equivalency. If resources permit, in the ideal situation sites should capture extended outcomes: mortality, NIHSS, and mRS at 90 days. Finally, we recommend active monitoring of transfer center data, to ensure continuous assessment of system-wide TeleNCC utilization and effects.

Several system, operational, and outcome specific metrics may require either specific adaptation for the individual site or cautionary interpretation. For example, value provided by a TeleNCC system is dependent on the intrinsic structure of how the provision of care is established, billed, and shared between sites. In addition, implementation and growth of a TeleNCC system correlates with increased admissions and local care for patients with worse disease severity, which often were previously transferred to the hub NCCU, and therefore the spoke outcomes may correspondingly become worse.

#### Rationale 12.1:

Improvement in NCCU outcomes include: reduction of in-hospital mortality, LOS, and greater odds of discharge to home [49–51]. Similar improvements are described with ICU telemedicine programs [52, 53]. While there is limited data on the effectiveness of TeleNCC [4], panelists agreed that with successful establishment of a TeleNCC program similar outcome improvements to in-person NCC should be attained. By instituting a neurointensivist, whether locally or through telemedicine, patients can receive similarly advanced neurocritical care management, which is supported by prior data. For several disease types, including large vessel ischemic stroke treated with endovascular thrombectomy, TeleNCC care of these patients has equivalent favorable functional outcomes to in-person care [7].

Change in outcomes attributed to in-person neurocritical care focuses on individual disease processes, which are equally relevant in TeleNCC. These are categorized into primary pathology categories: ischemic stroke, ICH, aSAH, and traumatic brain injury (TBI) [54]. The outcomes of decreased hospital and ICU LOS, decreased mortality, increased odds for improved discharge disposition, and rate of short- and long-term functional outcomes are most reported. Panelists stress that when considering mortality outcomes, care should be taken to whether the mortality was secondary to withdrawal of life sustaining therapy, an unanticipated death, or an expected death based on disease severity. Finally, to enhance safety and quality, measuring defined safety event rates was supported by all panelists. There should be quick recognition and evaluation of root causes to allow for creation and implementation of strategies for preventing such events in the future.

Longer term functional outcomes are valuable but are often challenging to measure. The most commonly used functional outcome for stroke and many other neurological diseases is the modified Rankin Scale (mRS). This ideally is measured at 90 days post injury [2]. The mRS is a simple and validated tool and is increasingly used in other disease states. Of note, ischemic stroke patients should have admission and discharge National Institutes of Health Stroke Scale (NIHSS) recorded. Admission metrics described in the quality section of this statement are key to determining the severity of illnesses cared for by TeleNCC and will help analyzing the long-term outcomes. The panelists recognize that a variety of longer-term outcomes may not be feasible for developing TeleNCC systems due to staffing and financial resources needed to capture the data. However, it was felt that when feasible, TeleNCC systems should aim to measure mRS on patients at 90 days postinjury.

The AHA/ASA Quality in Telestroke Statement similarly calls for the measurement of compliance with the aforementioned relevant and quantifiable processes [2]. While TeleNCC inherently cares for broader disease processes, it is reasonable to share in adoption of some of these benchmarks.

Finally, monitoring financial outcomes and benefits should be considered. The value TeleNCC may bring to each system was felt to be unique by panelists, often specific to their own system, and not generalizable to all systems of TeleNCC. Value driven outcomes are hence dependent on the type of financial model the systems have agreed to and operate within. For example, in a system where a RVU model is agreed to, monitoring the additional RVUs generated by the TeleNCC system may demonstrate that the additional income generated pays for the TeleNCC system outright. In other cases, the impact on bundled care payments relating to staffing costs for the hub versus spoke versus the entire system is relevant. Alternatively, if the model is based on a lump sum or “subscription fee” for continuous coverage of the spoke site with no additional billing, monitoring additional revenue by staffing costs or decreasing transfers may be helpful.

As a concluding consideration, it should be recognized that the initial adoption and growth of a TeleNCC system may have an unpredictable effect on patient outcomes and perception of value. In other words, a TeleNCC system newly covering a hospital may allow more patients to remain at the spoke and the case mix of such patients may favor sicker patients being retained more than previously. This has implications for increasing LOS and worse outcomes given the baseline clinical characteristic severity. Panelists articulated that therefore certain outcomes are more challenging to compare with pre-TeleNCC involvement, and that adjustment for comorbidity and disease severity may be necessary.

### Question 12.2: How Should Outcome Metrics be Analyzed and Reported?

#### Recommendation 12.2:

Outcome analysis should be done on a quarterly or semi-annual basis and the results reviewed with hub and spoke TeleNCC stakeholders. Quality improvement and implementation science methodology may be considered when approaching analysis results. It may be reasonable to use a predefined outcome questionnaire.

Outcomes should be reported by the site with the most experience in measuring outcomes with neurological injury; this typically is the hub site. Panelists suggest that quality leaders at institutions track all patients cared for by TeleNCC. Data storage and sharing should comply with national and local security regulations and be deidentified, when possible, for research and reporting.

#### Rationale 12.2:

The panelists suggest standardized and regular analysis and reporting of outcomes. Since most systems that utilize TeleNCC use an EHR, it was felt that data query and extraction for analysis can be semi-automated. Many systems also have access to an automated electronic dashboard that captures data for each TeleNCC encounter. If access to an electronic dashboard is not available, the TeleNCC system should consider using institutional-based discharge data that is provided within the EHR or billing system. Systems with a centralized transfer center may be able to track the number of consults and transfer rates, allowing easier visualization of system-wide data. Individual chart review may be necessary in some cases.

Several panelists commented that one approach to analysis and summative reporting could adopt some individual components of other existing quality outcome systems to help promote protocolization, effectiveness, and efficiency of care delivery to spoke sites. For example, the ASA/AHA “Get With Guidelines” for stroke care, which utilizes shared metrics for outcome reporting and achievement levels [55].

Whether outcomes are recorded at the hub or the spoke site is dependent on the relationship and capabilities of each site. There should be a standing, written agreement on who will be analyzing and measuring outcomes, with clear delineation of responsibilities. The responsibility of reporting outcomes usually is on the site with the most experience measuring these outcomes, which in most cases will be the hub site. Analysis and reporting of these outcomes done on a quarterly or semi-annual basis was felt by panelists to achieve balance of feasibility and turnaround time to address issues and implement changes.

Finally, TeleNCC is evolving, and the global use supports additional outcome analysis and pooling of data. It may be useful for research agreements between TeleNCC systems to share outcome data, then to be maintained within central research office, allowing for multi-institutional collaboration. Using a predefined, structured reporting form would allow for consistency in data and contribute to a more comprehensive understanding of TeleNCC impact on patient care. Last, analysis using artificial intelligence may help improve care and outcomes.

## Section 13: Research and Training

### Question 13.1: What are the Research Gaps in TeleNCC and What Should be Done to Address These Gaps?

#### Recommendation 13.1:

There is limited research on patient and system level effects, optimal format, and cost utility of TeleNCC programs. Future directions of research for TeleNCC should include rigorous, prospective randomized control trials addressing several questions (Table 7).

**Table 7:** TeleNCC domains, gaps in understanding, and select associated future research questions

Domain	Question
Patient assessment and evaluation	How does TeleNCC affect reliability and validity of the clinical assessment? How does TeleNCC affect the accuracy of the clinical diagnosis? How does TeleNCC affect the timeliness, efficacy, and effectiveness treatment plans?
Logistics and resources	What personnel and resources should TeleNCC include? How is a consultative TeleNCC service optimally staffed? To what extent does TeleNCC affect safety, harm, and ultimately morbidity and mortality, both in the ICU and afterwards? What is the (a) cost effectiveness and (b) total impact on healthcare utilization of different types of TeleNCC models for a spoke hospital, for a large hospital system, and if done in conjunction with other telemedicine services? Does a TeleNCC program affect the need for patient transfer to higher level of care centers?
Education and training	What training should TeleNCC providers receive and does formalized training affect care outcomes?
Patient care outcomes	How do patients and their families find quality of care TeleNCC compared to in-patient NCC? Are patient centric neurological outcomes being met? Does the extent of EHR integration impact care and outcomes?

#### Rationale 13.1:

Large research gaps in TeleNCC exist. With limited exception, the existing literature often lacks rigorous research designs. This is compounded by several barriers, such as financial cost of TeleNCC program implementation, technological ability to implement, availability of board certified neurointensivists, and willingness of potential facilities to participate in TeleNCC programs. Collectively, how TeleNCC programs deliver quality of care and the association and causal relationship to patient outcomes remains unclear. Furthermore, existing studies examining the use of TeleNCC are only inclusive of a few neurocritical care diseases, limiting the extrapolation of the benefit of TeleNCC to neurocritical care to the breadth of disease in neurocritical care. Prior studies show a spectrum of effect by TeleNCC as compared to in-person care, ranging from no benefit at all, to equivalency, to a trend toward benefit [5, 7, 12, 56].

### Question 13.2: To What Degree Should TeleNCC-Specific Training be Included for Critical Care Providers?

#### Recommendation 13.2:

Exposure to *a minimum* of some degree of TeleNCC and/or telemedicine experiential education should be included in fellowship training programs for all clinical providers who may be working in a neurocritical care setting. TeleNCC or telestroke rotations, standardized and monitored patient encounters, or patient didactics can be used to provide this fundamental skillset.

*In the optimal setting*, the panelists encourage training programs, particularly neurocritical care physician fellowships, to include TeleNCC rotations and didactics. For other providers, we suggest exposure to field-specific practice of telemedicine, or telecritical or neurocritical care if possible. Board certification organizations should support but not require TeleNCC into the optional components of the training.

The future of TeleNCC training may comprise an opportunity for earning certificates of special competence in TeleNCC if satisfactory completion of requirements is met. Training courses and workshops may be offered by high-volume TeleNCC institutions to expand educational opportunities. There are no such certificates or programs in existence currently.

#### Rationale 13.2:

Most formal training programs in telemedicine are in the form of postgraduate fellowships and are especially common in emergency medicine. Telemedicine has not traditionally been a standard feature of care educational programs in medicine, nursing, pharmacy, or other health disciplines. However, with the increasing use of TeleNCC as a solution to the growing needs for neurocritical care expertise, it is important that telemedicine education be provided, especially to TeleNCC providers. Many fundamental aspects of successful and safe TeleNCC practice are shared with other forms of telemedicine, especially telestroke, so experience and training in one of these may be sufficient for TeleNCC practice.

Given the early stages of TeleNCC use and frankly explosive expansion, specific certification requirements for training programs are not yet reasonable. Until standard practice TeleNCC has been adopted, as per this Consensus Statement, most training sessions on TeleNCC are focused on as opportunities rather than strict requirements.

Notably, in nursing, telemedicine educational opportunities include post baccalaureate certificate programs in telehealth nursing. Some states in the USA require nurses practicing in telehealth to receive formalized training to continue practicing in this environment (e.g., Washington State Department of Health, n.d.) and some RNs were eligible for the historical board certification in telecritical care nursing.

Finally, panelists felt that training providers to practice competently in a remote capacity via a telemedicine environment is important. There is an opportunity for specialty neuroscience-focused organizations to collaboratively devise TeleNCC certifications, however this subfield is not mature enough for such at present.

## Conclusions

TeleNCC may be an effective method to deliver neurocritical care in acute care environments lacking applicable in-person expertise provided the minimum requirements for standard practice and operations as listed in this document are met (see Table 8, summary and overview of recommendations)

**Table 8:** Overview of all TeleNCC recommendations contained in this Consensus Statement

**Models of TeleNCC**
*Recommendations 1.1: Models* The minimum TeleNCC model should be neurointensivist-directed and incorporate in-patient care interactions similar to that of an in-person neurointensivist. The hub TeleNCC provider for spoke sites provides consultative decision-support for the in-person primary provider via (a) telemedicine components and (b) access to raw medical data necessary for high level patient care, similar to an in-person provider. TeleNCC coverage should be offered 24/7/365 continuously, however it is reasonable to use only off-hour coverage (i.e., nights and/or weekends), so long as there are clear time-based coverage constructs of roles and responsibilities Some forms of practice do not meet minimum criteria for TeleNCC. These include telephone-only consultation, monitoring of neurological data feeds only without a standard practice for interactive care, or consultation without access to raw neurological data that would be typically required for critical care decision making
**Organization within the healthcare system**
*Recommendation 2.1: Operational needs and components* A needs assessment for TeleNCC in a healthcare system should encompass the characteristics of coverage needed. Coverage models may vary and generally include temporary off hours or short term, permanent, or variable to accommodate a spectrum of patient volumes The coverage should incorporate the parameters specified in the above needs assessment as well as those desired for meeting or exceeding the defined level of NCC coverage, Level 1–3, as described for NCC units
*Recommendation 2.2: Partnerships* Well-defined department roles and staffing partnerships are necessary for TeleNCC service function, in particular the on-site, in-person team(s): the primary ICU staff and the non-ICU neurology staff Specific scope of coverage for each provider should be defined, especially for covering non-neurological medical issues, procedures, and care before and after the ICU admission. Co-management team roles should be identified and agreed upon
*Recommendation 2.3: Measures of Success* The TeleNCC service or the host hub site should capture and track metrics for both system impact as well as for patient care and outcomes
**Required and optional structural elements**
*Recommendations 3.1A: Minimum requirements* TeleNCC must meet or exceed the benchmarks (below) for: telemedicine technology interface, involved personal, core components of the consult, and documentation. The benchmarks: 1. Technology and telecommunication infrastructure must have reliable high-speed internet connectivity for videoconferencing, data transmission, and remote monitoring 2. Advanced imaging capabilities should allow radiologic studies to be viewable by the hub provider via a remote portal or be integrated into the hub imaging system 3. Electronic health record (EHR) integration should allow seamless access to medical history, imaging studies, laboratory results, and other relevant clinical information 4. Data security and privacy measures must be in place to ensure integrity and protection of patient health information (PHI). These should adhere to relevant local and national regulations, such as the Health Insurance Portability and Accountability Act (HIPAA) in the USA 5. Trained personnel for providing hub TeleNCC encompasses: neurocritical care physicians, advanced practice providers, nurses, pharmacists, and technical support staff who are proficient in using the telemedicine technology. The spoke team must have an onsite primary clinical provider who can effectively collaborate remotely with the hub consultative team 6. Clinical documentation should be clear, concise, and accurate as per typically required for patient care, communication, and legal compliance General guiding telemedicine privileges and documentation standards for hub TeleNCC providers are presented in Tables 1 and 2
*Recommendations 3.1B: Additional, optimal elements* In well-resourced settings additional, advanced TeleNCC components include: dedicated staffing, a spoke TeleNCC APP and/or RN who have assigned hours or percent of their FTE to TeleNCC. It is optimal to have a backup two-way audiovisual cart or robot or other alternative communication device in case of primary technology failure
**Staffing and engagement of qualified multidisciplinary teams**
*Recommendations 4.1: Levels of staffing at hub* Staffing should be provided by qualified TeleNCC care providers, as demonstrated by applicable residency and fellowship training and board-certification or board-eligibility in neurocritical care for physicians, or appropriate training and credentialing for other types of providers
*Recommendations 4.2: Staffing at spoke* Each spoke should have a dedicated individual (site champion) who is responsible for providing training and supervision for other medical providers and staff members utilizing TeleNCC services At a minimum, providers who interface with TeleNCC providers at participating sites should receive adequate training in the relevant telemedicine and telecommunication technologies, TeleNCC workflows, policies and procedures, and key clinical assessments. A provider capable of performing bedside critical care procedures, such as airway management and central line placement, and responding to emergencies in-person must be available in-person at each participating site
**Roles and operational responsibilities for specific provider types**
*Recommendation 5.1: Of the team providing TeleNCC* The composition will vary according to the level of services required and the TeleNCC model that is adopted—at a minimum, it is a solo NCC physician, and at the other end of the spectrum, the team may be comprised of a combination of health care practitioners including a NCC physician, a critical care RN, a neurocritical care APP, a NCC fellow, and a pharmacist Specific responsibilities for each provider are listed in the main text and Table 3
**Activation and communication**
*Recommendation 6.1: Methods of communication:* A TeleNCC team may carry a dedicated phone or single contact phone number that forwards all calls to the provider on duty, allowing direct access to the consulting neurointensivist. Alternatively, they may be contacted via phone call, text, or paging system or mobile app, through an on-call provider listing, transfer center, or some combination of these
*Recommendation 6.2: Activation of a consult* The panelists recommend that activation of a consult should be via beeper/pager or phone with response time to triage the consult in less than 10–15 min. The time to initiate the actual consult request will vary depending on the urgency of the initial request, and initial triage conversations are suggested to prioritize multiple consults It is important to establish clear expectations regarding initial communication between the teleneurointensivist and on-site primary teams, including what information is needed when activating a consult, who activates the consult, which types are covered between TeleNCC and telestroke (if available), and how quickly a response from the TeleNCC team should be expected
*Recommendation 6.3: Follow-up after consult* Once the two-way audio-visual TeleNCC evaluation has been conducted, clear recommendations should be communicated to the spoke hospital consulting team, both verbally and via documentation in a summary consultative note contained in the EHR
*Recommendation 6.4: Ongoing communication* It is important to maintain clear communication channels between spoke teams and TeleNCC, and also between TeleNCC providers. When follow up questions arise, outside of regularly planned rounding times, it is important to establish if and how TeleNCC teams can be reached
**Afterhours and Anticipatory guidance**
*Recommendation 7.1: Availability* TeleNCC programs should be prepared to provide access 24 h a day, 7 days a week, 365 days a year. Programs that are unable to provide continuous 24/7 coverage should ensure that an alternative service with appropriately trained and credentialed providers is available to fill any gaps in coverage
*Recommendations 7.2: Anticipatory guidance* When providing consultative support, TeleNCC providers should make every effort to include contingencies for an appropriate range of anticipated and unanticipated but possible clinical trajectories
*Recommendation 7.3: Family and surrogate engagement* Each TeleNCC system establishes a system by which patients and their families have access to direct interaction with TeleNCC providers
**Regulatory and credentialing**
*Recommendation 8.1: Interstate* Measures to reduce the administrative burden of obtaining incremental licenses should be fully supported, including the adoption and diffusion of licensing compacts, where not yet enacted. As an additional measure, state jurisdictions are encouraged to promulgate telehealth registration processes to further expedite out-of-state clinicians obtaining authorization to practice telehealth
*Recommendation 8.2: Credentialing by proxy (CBP)* CBP arrangements, which allow the spoke hospital to recognize the credentialing measures by the hub hospital, should be pursued whenever amenable to the spoke site. The spoke site should retain the ability, contractually, to trigger full credentialing measures on an as-needed basis or subject to specific conditions
*Recommendation 8.3: Credentialed to specific hospital* The TeleNCC provider should be CBP to the spoke site’s hospital. The spoke site should define the appropriate scope of access for providers credentialed under CBP measures There should be a minimal, standardized amount of required training for spoke EHR access, as the provider both abides by HIPAA regulations and should be compliant with local security and EHR usage training at their hub institution
*Recommendation 8.4: Risk mitigation* Prior to the implementation of TeleNCC consultations, contractual agreements must be in place to define the scope of the service to be performed The agreement should define duration and term of the arrangement, applicable statutory and regulatory requirements, and the expected obligations and scopes of work for each party to the agreement The agreement may also include protocols for participation quality improvement and after-action review(s), on an as-needed basis if desired by the parties
**Finance**
*Recommendations 9.1: Financial reimbursement* Hub: For TeleNCC video services, the billing type will vary based on the organization of the health system and the associated contractual agreements. In most cases, the provider should bill a telemedicine specific code (detailed in main text). Alternatively, the provider may not directly bill themselves per encounter when the contract is based on per click consultation rates, with or without a base contractual reimbursement In some circumstances, when possible, the provider may bill as the In-Person Equivalent CPT code as if the patient was seen in a healthcare facility (i.e. Facility to Facility) Spoke: For TeleNCC video services, the spoke site may be able to bill a technical facility charge (i.e. Q3014). The availability of this facility fee will vary by payer Additional recommendations on cost estimation and data capture, as well as specific billing codes for type of care are listed in the main text and Table 4
**Pharmacy**
*Recommendation 10.1A: Minimum requirements* Clinical pharmacist(s) are available for, or included in, spoke multidisciplinary or hub teleneurocritical care team rounds. This can be conducted in-person or virtually, via telemedicine remote review and management. A standard means and time of communication should be established to ensure consistency and limit variation. Additional detail in Table 5
*Recommendation 10.1B: Optimal requirements* The clinical pharmacist working with the TeleNCC team should be a dedicated neurocritical care pharmacist, with critical care specialist training, and ideally board-certification in critical care pharmacotherapy (BCCCP) A critical care pharmacist is available 24 h and has dedicated NCC/TeleNCC time in their FTE, be included in multidisciplinary rounds for TeleNCC patients
*Recommendation 10.2: Operational components* Pharmacy services at the spoke facility must ensure the safe procurement, compounding, delivery, and administration of all medications
**Quality**
*Recommendation 11.1: Quality metrics* In addition to specific metrics of telecommunication integrity and quality, TeleNCC systems should capture consult volume with time per encounter and characterize the similar quality metrics of daily operations as compared to an in-person NCCU. Additional details in Table 6
**Outcomes**
*Recommendation 12.1: Outcome metrics for TeleNCC effect:* The following outcomes should be measured: transfer rate from the spoke to the hub, adverse events (examples below, in rationale), hospital and ICU length of stay (LOS), in-hospital mortality, discharge disposition, and National Institutes of Stroke Scale (NIHSS) and modified Ranking Scale (mRS) at discharge. At a minimum, the transfer rate from spoke to the hub site, mortality, and discharge metrics of NIHSS when appropriate and mRS should be compared to in-person NCCUs for equivalency. Additional details in Table 6
*Recommendation 12.2: Review and reporting of metrics* Outcome analysis should be done on a quarterly or semi-annual basis and the results reviewed with hub and spoke TeleNCC stakeholders
**Research and training opportunities**
*Recommendations 13.1: Research gaps* There is limited research on patient and system level effect, optimal format, and cost utility of TeleNCC programs. Future directions of research for TeleNCC should include rigorous, prospective randomized control trials addressing several questions. Additional details in Table 7
*Recommendations 13.2: Training in TeleNCC* Exposure to a minimum of some degree of TeleNCC and/or telemedicine experiential education should be included in fellowship training programs for all clinical providers who may be working in a neurocritical care setting

## Abbreviations

TeleNCC: Teleneurocritical care
NCCU: Neurocritical care unit
ED: Emergency department
ICU: Intensive care unit
TCC: Telecritical care
NCS: Neurocritical care society
AHA: American Heart Association
ASA: American Stroke Association
ATA: American Telemedicine Association
NCC: Neurocritical care
ICH: Intracranial hemorrhage
aSAH: Aneurysmal subarachnoid hemorrhage
EHR: Electronic health record
EEG: Electroencephalogram
HIPAA: Health Insurance Portability and Accountability Act
PACS: Picture archiving and communication system
PHI: Protected health information
NP: Nurse practitioner
PA: Physician assistant
APP: Advanced practice provider
RN: Registered nurse
RT: Respiratory therapist
ENLS: Emergency neurological life support
RCA: Root cause analysis
QI: Quality improvement
TCD: Transcranial doppler
CBP: Credentialed by-proxy
BCCCP: Board certification in critical care pharmacotherapy
COP: Conditions of participation
LOS: Length of stay
TBI: Traumatic brain injury
mRS: Modified Rankin Scale
NIHSS: National Institutes of Health Stroke Scale
